# Metabolite pools and carbon flow during C_4_ photosynthesis in maize: ^13^CO_2_ labeling kinetics and cell type fractionation

**DOI:** 10.1093/jxb/erw414

**Published:** 2016-11-07

**Authors:** Stéphanie Arrivault, Toshihiro Obata, Marek Szecówka, Virginie Mengin, Manuela Guenther, Melanie Hoehne, Alisdair R Fernie, Mark Stitt

**Affiliations:** Max Planck Institute of Molecular Plant Physiology, Am Muehlenberg, Potsdam-Golm, Germany

**Keywords:** ^13^C labeling, C_4_ photosynthesis, carbon flow, CO_2_-concentrating shuttle, maize

## Abstract

Worldwide efforts to engineer C_4_ photosynthesis into C_3_ crops require a deep understanding of how this complex pathway operates. CO_2_ is incorporated into four-carbon metabolites in the mesophyll, which move to the bundle sheath where they are decarboxylated to concentrate CO_2_ around RuBisCO. We performed dynamic ^13^CO_2_ labeling in maize to analyze C flow in C_4_ photosynthesis. The overall labeling kinetics reflected the topology of C_4_ photosynthesis. Analyses of cell-specific labeling patterns after fractionation to enrich bundle sheath and mesophyll cells revealed concentration gradients to drive intercellular diffusion of malate, but not pyruvate, in the major CO_2_-concentrating shuttle. They also revealed intercellular concentration gradients of aspartate, alanine, and phosph*enol*pyruvate to drive a second phospho*enol*pyruvate carboxykinase (PEPCK)-type shuttle, which carries 10–14% of the carbon into the bundle sheath. Gradients also exist to drive intercellular exchange of 3-phosphoglycerate and triose-phosphate. There is rapid carbon exchange between the Calvin–Benson cycle and the CO_2_-concentrating shuttle, equivalent to ~10% of carbon gain. In contrast, very little C leaks from the large pools of metabolites in the C concentration shuttle into respiratory metabolism. We postulate that the presence of multiple shuttles, alongside carbon transfer between them and the Calvin–Benson cycle, confers great flexibility in C_4_ photosynthesis.

## Teaser

Analysis of labeling kinetics, pool sizes, and concentration gradients of metabolites reveals the operation of multiple decarboxylation pathways and rapid movement of carbon between the Calvin–Benson cycle and the CO_2_-concentrating shuttles in maize.

## Introduction

The CO_2_-concentrating shuttle (CCS) in C_4_ photosynthesis requires close co-operation between mesophyll cells (MCs) and bundle sheath cells (BSCs) (Hatch and Slack, 1966; Hatch and Osmond, 1976; Weber and von Caemmerer, 2010). CO_2_ is initially incorporated into oxaloacetate (OAA) in the MCs by phosph*enol*pyruvate carboxylase (PEPC), whose high affinity allows it to operate at low CO_2_ concentrations. OAA is converted to other four-carbon metabolites that move to the BSCs and are decarboxylated, generating a high CO_2_ concentration that allows efficient operation of the Calvin–Benson cycle (CBC), and three-carbon metabolites return to the MCs. C_4_ plants have historically been classified into three subtypes based on the major decarboxylation enzyme (Hatch and Osmond, 1976; Weber and von Caemmerer, 2010; Supplementary Fig. S1 at *JXB* online): the NADP-malic enzyme (ME) subtype where malate moves to the BSCs and pyruvate returns to the MCs; the NAD-ME subtype where aspartate moves to the BSCs and alanine returns to the BSCs; and the phospho*enol*pyruvate carboxykinase (PEPCK) subtype where NAD-ME also contributes to decarboxylation. and aspartate and malate move to the BSCs, and PEP and alanine move back to the MCs. In some cases the shuttles also transfer energy to the BSCs, for example NADPH in the NADP-ME subtype. In many NADP-ME species, including maize, PSII in the BSCs is strongly decreased and ATP and NADPH are supplied to the BSCs via another intercellular shuttle; 3-phosphoglycerate (3PGA) moves from the BSCs to the MCs where it is reduced by phosphoglycerate kinase and NADP-glyceraldehyde-3-phosphate dehydrogenase to triose-phosphate (triose-P) which move back to the BSCs. The topology of C_4_ photosynthesis was established in the 1970–1980s but fundamental questions remain concerning its operation.

One issue relates to how metabolites move between the MCs and BSCs. It was proposed in the 1970s that this occurs by diffusion (Hatch and Osmond, 1976). Diffusion will be facilitated by the high density of plasmodesmata and close vein spacing which means that each MC is adjacent to a BSC (Evert *et al.*, 1977; Nelson, 2011; Fouracre *et al.*, 2014). Rapid diffusion requires concentration gradients between the MCs and BSCs, estimated in the range of 5–10 mM (Hatch and Osmond, 1976). In the 1980s, two methods were developed to enrich MCs and BSCs from maize leaves while preventing changes in metabolite levels (Flügge *et al.*, 1985; Leegood, 1985; Stitt and Heldt, 1985*a*, *b*). Both reported gradients to support malate and triose-P movement from the MCs to the BSCs, and 3PGA movement from the BSCs to the MCs, but not for pyruvate. However, it remained unclear what proportion of the overall pools were actually involved in photosynthesis, as opposed to being located in, for example, the vacuole or other cell types.

Secondly, it may be overly simplistic to classify C_4_ plants into three subtypes (Furbank, 2011; Bellasio and Griffiths, 2014; Wang *et al.*, 2014*b*). There is evidence for parallel operation of shuttles, including co-occurrence of enzymes and high contents and rapid labeling of diagnostic metabolites. However, part or most of the metabolite content may not be involved in C_4_ photosynthesis (Stitt and Heldt, 1985*b*; Wang *et al.*, 2014*a*) and labeling might be due to label exchange rather than net flux through the metabolite.

Thirdly, other metabolic pathways must be modified to make them compatible with the high concentrations of metabolites that are involved in intercellular shuttles (Bräutigam *et al.*, 2014). One example is sucrose synthesis, which occurs in the MC cytosol (Furbank *et al.*, 1985). The affinity of maize cytosolic fructose bisphosphatase is an order weaker than that of the C_3_ enzyme, allowing high concentrations of triose-P to be maintained in the MCs to drive diffusion to the BSCs (Stitt and Heldt, 1985*a*). It is not known how the high concentrations of organic and amino acids are shielded from respiration (Bräutigam *et al.*, 2014).

A fourth question concerns interactions between the CCS and the 3PGA/triose-P shuttle. Interconversion of PEP and 3PGA will allow carbon to move between these shuttles, and ultimately into or out of the CBC. It has been proposed that large pools of organic acids and amino acids buffer CBC metabolite levels (Leegood and Furbank, 1984; Leegood and von Caemmerer, 1989). However, it is not known how rapidly 3PGA and PEP are interconverted, and how movement of carbon from one shuttle to the other is regulated. Shuttle operation depends on high metabolite concentrations to drive diffusion, and efficient C_4_ photosynthesis depends on a correct balance between the rate at which carbon is shuttled into the BSC and its utilization by the CBC (de Veau and Burris, 1989; von Caemmerer and Furbank, 2003; Bellasio and Griffiths, 2014).

It is also unclear how far photorespiration is decreased in C_4_ plants. Compared with C_3_ plants, C_4_ plants have lower but still substantial activities of enzymes for photorespiration (Osmond and Harris, 1971; Ohnishi and Kanai, 1983; Ueno *et al.*, 2005). Evidence for the occurrence of photorespiration has been provided by investigating O_2_ dependency (Dai *et al.*, 1993, 1995; Maroco *et al.*, 1998) and ^18^O incorporation (de Veau and Burris, 1989; Lawlor and Fock, 1978), and a comparative study in the *Flaveria* genus revealed 2- to 5-fold lower photorespiration in C_4_ than in C_3_ species (Mallmann *et al.*, 2014). Further, photosynthesis is impaired in maize mutants with decreased glycolate oxidase activity (Zelitch *et al.*, 2009).

Labeling studies with ^14^CO_2_ played a key role in elucidating C_4_ photosynthesis (Slack and Hatch, 1967; Hatch, 1971, 2002; Nickell, 1993). In recent years, analysis of labeling kinetics in individual metabolites has been greatly facilitated by the use of stable isotopes in combination with GC-MS or tandem liquid chromatography (LC-MS/MS) (Antoniewicz *et al.*, 2006; Schwender *et al.*, 2006; Yuan *et al.*, 2006; Huege *et al.*, 2007; Hasunuma *et al.*, 2009). We recently established a protocol for dynamic ^13^CO_2_ labeling of whole Arabidopsis rosettes, analyzed the temporal labeling kinetics of ~40 metabolites using GC-MS and LC-MS/MS, estimated fluxes, and benchmarked them against literature values (Szecowka *et al.*, 2013). ^13^CO_2_ labeling also provides information about the sizes of pools that are involved in photosynthesis; in many cases, only part and sometimes only a minor part of the total pool is directly involved (Szecowka *et al.*, 2013; Ma *et al.*, 2014). We now adapt this method to analyze C_4_ photosynthesis in maize to address the five questions discussed above. In addition, we analyze ^13^C labeling patterns in fractions enriched in MCs and BSCs, to determine the intercellular distributions and concentration gradients of the metabolic pools that are involved in C_4_ metabolism, and provide independent evidence for operation of more than one CCS.

## Materials and methods

### Chemicals

Carbon dioxide (^13^CO_2_, isotopic purity 99 atom%) was from Campro Scientific, and chemicals from Sigma-Aldrich, Roche, or Merck.

### Plant growth

Maize (*Zea mays* L. cv. B73) seeds were germinated in darkness in Petri dishes on moistened filter paper (3 d, 28 °C), transferred to soil in 10 cm diameter pots, grown for 5 d under 16/8 h day/night cycles (irradiance 105 µmol photons m^−2^ s^−1^, 22/18 °C, 70% relative humidity) and then under 14/10 h day/night cycles (irradiance 480 µmol photons m^−2^ s^−1^, 25/22 °C, 65% relative humidity) and used at 3 weeks for ^13^CO_2_ labeling. *Arabidopsis thaliana* Col-0 was grown as in Szecowka *et al.* (2013). CO_2_ concentration in our growth facilities was ~420 ppm.

### ^13^CO_2_ labeling and quenching procedure for the maize kinetics experiment

Gas was mixed to a final concentration of 78% N_2_, 21% O_2_, and 420 ppm ^12^CO_2_/^13^CO_2_, humidified, pumped into a custom-designed labeling chamber, and exited via a PVC tube over soda lime to capture ^12^CO_2_/^13^CO_2_ [Supplementary Fig. S2A, B; see Szecowka *et al.* (2013)]. The chamber (volume 320 ml) had a gas half-life of 2.6 s and 1.3 s at flow rates of 5 l min^−1^ and 10 l min^−1^, respectively. Flow at 10 l min^−1^ was used for pulses of up to 1 min, and 5 l min^−1^ for longer pulses. The chamber was made of copper with a hollow body and a transparent Plexiglas lid with a hollow vertical tube (internal diameter ~2 cm) sealed with transparent film (Toppits^©^). The lid was fastened using clasps, with a soft rubber O-ring between the lid and chamber, ensuring air-tight closure. An additional lamp (FL-460 Lighting Unit, Walz, Effeltrich, Germany) was placed beside the labeling chamber to ensure an irradiance in the chamber of 480 µmol photons m^−2^ s^−1^. To obtain a constant leaf temperature of 35 °C (measured in the growth chamber with a thermocouple; VWR and Testo), gas was passed through a humidifier in a water bath (gas relative humidity of 65%), and water was pumped through the hollow body of the labeling chamber. Plant material was quenched by dropping a pre-cooled (in liquid N_2_) copper rod with a sharp machined edge down the hollow tube in the lid into the labeling chamber, freeze-clamping a leaf disc (1.9 cm diameter, ~65 mg FW) between this rod and another copper rod fixed in the chamber, protruding into the base of the chamber and extending well below the outside of the chamber to allow pre-cooling with liquid N_2_ (Supplementary Fig. S2C, D). Leaf four was used after it reached full expansion, when plants were ~3 weeks old. Labeling started 2 h after the start of the light period to ensure metabolic steady state. The leaf was placed in the labeling chamber with a continuous flow of 78% N_2_, 21% O_2_, and 420 ppm ^12^CO_2_ (Supplementary Fig. S2A, B), and after 1 min ^12^CO_2_ was replaced by ^13^CO_2_. Samples were collected 10, 15, 20, 30, and 50 s, and 1, 3, 5, 10, 20, 40, or 60 min after starting labeling, in a random manner. Unlabeled samples (*t*=0) were collected after 1 min in an unlabeled gas mixture. Leaves were also labeled for 60 min and chased with unlabeled gas for 5 min or 20 min. Leaf discs from two plants were pooled per time point.

### ^13^CO_2_ labeling and quenching procedure for maize cell separation

To obtain material for cellular separation, ^13^CO_2_ air was applied in a transparent plastic oven bag (Pely-plastic, Wahlstedt, Germany) sealed at both ends with bag ties/clasps (Supplementary Fig. S2E). Gas entered and exited via PVC tubes inserted at the leaf base and the other end of the bag, respectively. Gas mixing, humidification, and irradiance were as described above. Labeling started at least 2 h after the start of the light period. The leaf was subjected for 1 min to a continuous flow (5 l min^−1^) of 78% N_2_, 21% O_2_, and 420 ppm ^12^CO_2_, and the flow then diverted for 1 min from the labeling chamber into the CO_2_ trap (Supplementary Fig. S2E), ^12^CO_2_ replaced by ^13^CO_2_ in the gas mixture, unlabeled gas removed from the bag by vacuum (Supplementary Fig. S2F), and the labeled gas mix then switched to the bag. Three minutes later the central leaf part (~500 mg FW) was quenched by clamping the leaf and bag between two aluminum blocks pre-cooled in liquid N_2_ (Supplementary Fig. S2G, H). Six leaves were harvested, and pooled in pairs to provide three biological samples.

### ^13^CO_2_ labeling and quenching for Arabidopsis

Arabidopsis rosettes were labeled and quenched as in Heise *et al.* (2014), except that they were subjected to 78% N_2_, 21% O_2_, and 420 ppm ^13^CO_2_ (ambient O_2_) or 97% N_2_, 2% O_2_, and 420 ppm ^13^CO_2_ (low O_2_). Plants were placed in a transparent box and provided (5 l min^−1^) with 420 ppm ^12^CO_2_ and either 78% N_2_/21% O_2_ or 97% N_2_/2% O_2_ for at least 30 min prior to switching to 420 ppm ^13^CO_2_, and sampled after 5, 10, 20, 30, and 45 s, and 1, 1.5, 2, 3, 5, 10, 20, 40, and 60 min, with 3–13 replicates per time point. Unlabeled samples (*t*=0) were collected after at least 30 min in ambient or low O_2_.

### Metabolite analyses and calculation of total pool size, enrichment, isotopomer distributions, positional enrichment, and ^13^C amounts

Maize or Arabidopsis material was ground to a fine powder by hand in a mortar pre-cooled with liquid N_2_ or in a cryo-robot (Stitt *et al.*, 2007), and stored at –80 °C. Samples were analyzed by LC-MS/MS and GC-MS, with authentic standards for accurate metabolite quantification, as in Heise *et al.* (2014). We additionally analyzed aspartate, PEP, 2-phosphoglycolate (2PG), ribose-5-phosphate (R5P), and ribulose-5-phosphate+xylulose-5-phosphate (Ru5P+Xu5P) [see Supplementary Tables S1, S2 for the isotopomer-dependent MS parameters used for selected reaction monitoring (SRM) and the corconfig.cfg file used to correct for natural abundance; Heise *et al.*, 2014; Huege *et al.*, 2014]. Amounts of the unlabeled form and each ^13^C isotopomers in maize samples are provided in Supplementary Table S3 and total contents in Supplementary Table S4. Total amounts of 3PGA and PEP were determined enzymatically using a Sigma-22 dual-wavelength photometer (Merlo *et al.*, 1993) using freshly prepared extracts for PEP. ^13^C Enrichment and isotopomer distribution were calculated as in Szecowka *et al.* (2013) (Supplementary Tables S5, S6). Active and inactive pools were calculated as in Supplementary Table S3, positional ^13^C enrichment (C4 and C1–C3 positions) of aspartate and malate as in Supplementary Tables S7, S8, and ^13^C amounts in metabolites as in Supplementary Tables S7, S8.

### Maize cell separation

Maize leaves were fractionated as in Stitt and Heldt (1985*a*, *b*). Four fractions were obtained by homogenizing ~1 g FW of material at low temperature, resuspending in liquid N_2_, and filtering sequentially through 200, 80, and 40 µm nylon meshes (Sefar, Switzerland). Activities of the MC markers NADP-malate dehydrogenase (NADP-MDH) and PEPC were measured as in Gibon *et al.* (2004) with 2000- and 20 000-fold dilution (FW/extract volume), respectively. The BSC marker ribulose-1-5-bisphosphate (RuBP) was quantified by LC-MS/MS. Cell separation was performed on three biological replicates, and these were combined to calculate distribution. The intercellular distribution of a metabolite X was estimated by extrapolation. Enzyme activities and metabolite amounts in each fraction were expressed as a percentage of that in the summed fractions. For each fraction, the ratio of (average of NADP-MDH and PEPc)/RuBP; *x*-axis) and the ratio (metabolite X/RuBP; *y*-axis) were plotted against each other, and regression calculated. The intercept on the *y*-axis represented the proportion of metabolite X in the BSCs. Distribution was calculated for each isotopomer, the sum of all isotopomers of a metabolite and, for malate, the sum of labeled isotopomers.

### Gas exchange

Net CO_2_ assimilation (*A*_n_) was measured on the fouth fully expanded leaf in a gas exchange system (LiCor) attached to a Leaf Chamber Fluorometer under similar conditions to the labeling experiment (480 µmol m^−2^ s^−1^ irradiance, 35 °C, 420 ppm CO_2_). *A*_n_ was measured first at 21% O_2_ and then at 2% O_2_ in six plants with eight technical replicates per plant.

## Results

### Temporal kinetics of ^13^C accumulation in metabolites in maize leaves

Leaves were pulsed with ^13^CO_2_ for 0, 10, 15, 20, 30, and 50 s, and 1, 3, 5, 10, 20, 40, and 60 min, or were pulsed for 60 min followed by a 5 min or 20 min chase with ^12^CO_2._ The labeling chamber was designed to have a small volume to minimize the time delay between introduction and full equilibration of ^13^CO_2_ and to allow leaf material to be quenched instantaneously under the prevailing light regime, and without allowing access of unlabeled CO_2_ for even a fraction of a second. The latter is important because many metabolites turn over very rapidly during photosynthesis (Arrivault *et al.*, 2009; Heise *et al.*, 2014). A total of 35 metabolites were detected with GC-MS and LC-MS/MS including all C_4_ photosynthetic intermediates, almost all CBC intermediates, intermediates in starch and sucrose biosynthesis and photorespiration, and organic acids and amino acids. The amount of the unlabeled form and each ^13^C isotopomer (Supplementary Table S3) were used to estimate the total amount (Supplementary Table S4), ^13^C enrichment (% of C atoms that are labeled with ^13^C; Supplementary Table S5), and relative abundance of each isotopomer (Supplementary Table S6) for each metabolite and time point.

Total metabolite content showed some variation (Supplementary Table S4). This was not related to the time of sampling (Supplementary Fig. S3). Variation in metabolite contents was seen previously between maize leaves (Leegood and von Caemmerer, 1988) and along the leaf developmental gradient (Pick *et al.*, 2011; Wang *et al.*, 2014*a*). The pulse and chase experiments were performed on different days. Metabolite contents were in the same range in both experiments except that glucose and fructose were higher, and pyruvate and fumarate were lower in the chase experiment (Supplementary Table S4). Total content was estimated as the average of all samples from the pulse and chase, unless stated otherwise.

### Overview of the temporal kinetics of ^13^C accumulation in metabolite classes

We subjected the ^13^C enrichment kinetics of all 35 metabolites to *k*-means clustering based on Euclidian distance (Supplementary Fig. S4, note log_10_ time scale). Based on the ‘rule of thumb’ (Mardia *et al.*, 1979), we generated four clusters. Cluster I contained metabolites whose enrichment was high (average 25%) within 10–30 s and were almost fully labeled (average 93%) after 40–60 min. It included most CBC intermediates, the starch biosynthesis intermediate ADPG, and the immediate product of RuBP oxygenation, 2PG. Cluster II contained metabolites that were labeled in the first 10–30 s although less strongly (average 11%) than cluster I, and whose enrichment rose to an average of 82% after 40–60 min. It included sucrose synthesis intermediates (G6P, G1P, and UDPG) and some metabolites involved in the CCS (PEP, aspartate). Cluster III showed very low enrichment (<1%) in the first 30 s, and rose gradually to an average value of 76% after 40–60 min. It included metabolites from the CCS (pyruvate, alanine) and photorespiration (glycine, serine). Cluster IV showed negligible labeling in the first 30 s and rose slowly to an average of 16% after 40–60 min. It included glycerate, which is the last metabolite in the photorespiration pathway, sugars, tricarboxylic acid cycle (TCA) intermediates [fumarate, succinate, 2-oxoglutarate (2OG)], many amino acids, and, unexpectedly, malate and sedoheptulose-1,7-bisphosphate (SBP).

The ^13^C enrichment kinetics for most metabolites in and downstream of the CBC resembled those of Arabidopsis (Szecowka *et al.*, 2013), showing that many features of C_3_ pathway topology are conserved in maize. However, there were some anomalies. First, metabolites involved in CCS such as malate, aspartate, pyruvate, alanine, and PEP were spread across three clusters (II, III, IV) and labeled more slowly than CBC intermediates. Indeed, malate was assigned to cluster IV. Secondly, the CBC intermediate SBP was assigned to cluster IV. Photorespiratory intermediates were also distributed across three clusters (I, III, IV). We considered two possible reasons for these anomalies. One is the presence of compartmented pools that are not involved in photosynthesis. The second is related to the topology of C_4_ photosynthesis (see below).

### Deconvolution of the overall labeling kinetics by considering inactive pools

**Fig. 1.**
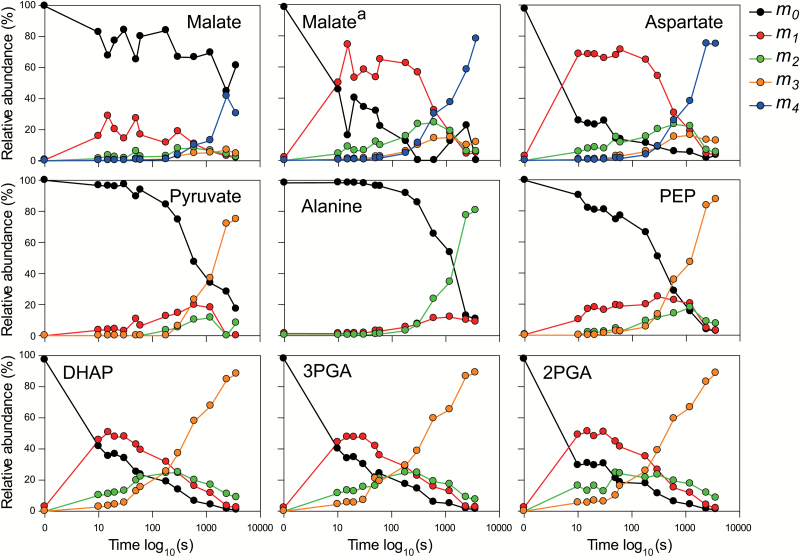
Time-course of mass distribution of metabolites involved in the shuttle concentrating CO_2_ in the BSCs. The relative abundance of each isotopomer (*m*_*n*_) for a given metabolite is represented; *n* is the number of ^13^C atoms incorporated. The graph for malate^a^ corresponds to the isotopomer distribution after correction for the inactive pool. The *x*-axis corresponds to the labeling time on a log_10_ scale. Data are presented in Supplementary Table S6.

When most or all of the total content of a metabolite is involved in photosynthesis, the unlabeled isotopomer will decrease to very low values and the fully labeled isotopomer will rise to become the predominant form. This pattern was seen for all CBC metabolites (Supplementary Fig. S5; Supplementary Table S6) except SBP, where >80% was present as the unlabeled form even at 60 min while the remainder was present as a mix of isotopomers with heavily labeled forms predominating. Similarly, whereas at 60 min the unlabeled form of PEP, aspartate, pyruvate, and alanine decreased to negligible levels and the fully labeled form predominated, ~60% of the total malate pool was unlabeled while most of the rest was the quadruple-labeled (i.e. fully labeled) isotopomer (Fig. 1). This pattern points to there being kinetically separated pools; an ‘active’ pool that is involved in photosynthetic flux and an ‘inactive’ pool that does not participate in photosynthesis (Szecowka *et al.*, 2013).

To estimate the active pool size of malate (malate^a^), we first summed the labeled isotopomers of malate [termed Malate(L)]. Compared with the 4-fold variation in total malate content (1.8–7 µmol g^−1^ FW; Supplementary Table S4), Malate(L) was fairly constant from 1 min onwards (1.1–1.7 µmol g^−1^ FW; Supplementary Table S3). Malate(L) at 60 min (1.3 µmol g^−1^ FW) was similar to the average of Malate(L) between 10 min and 60 min (1.29 µmol g^−1^ FW). The inactive pool of malate (Malate^i^) at each time point was estimated by subtracting 1.3 µmol g^−1^ FW from the total malate amount at that time (Supplementary Table S3). Malate^i^ was then subtracted from Malate-0 to provide an estimate of the amount of ^12^C malate in the active pool at each time (Malate-0^a^, Supplementary Table S3). Malate-0^a^ and the values for the labeled isotopomers were used to calculate ^13^C enrichment (Supplementary Tables S5) and relative isotopomer abundance (Fig. 1; Supplementary Table S6) for the active malate pool. The corrected isotopomer abundance profile (Malate^a^, Fig. 1) resembled that of aspartate although with noise, probably due to assumptions in estimating the active pool. A similar correction was performed for SBP (Supplementary Fig. S5; Supplementary Tables S3, S5, S6).

### Deconvolution of labeling kinetics based on the topography of C_4_ photosynthesis

PEPC incorporates bicarbonate into the C_4_ position of metabolites such as malate and aspartate, and carbon at the C_4_ position is released as CO_2_ in the BSCs (Supplementary Fig. S6A; Hatch and Slack, 1966; Hatch and Osmond, 1976; Moore and Edwards, 1986). Thus, early in the labeling kinetics, single-labeled isotopomers of malate and aspartate should dominate and there should be little label in decarboxylation products such as pyruvate and alanine. The appearance of multiple labeled isotopomers of malate and aspartate and labeling of pyruvate and alanine will be delayed until ^13^C moves from the CBC into PEP and other CCS metabolites (Supplementary Fig. S6B).

The isotopomer patterns for malate, aspartate, pyruvate, and alanine qualitatively matched these expectations. Single-labeled isotopomers of malate^a^ and aspartate were dominant at early time points (Fig. 1). For malate^a^, 50–74% was present as a single-labeled isotopomer at 10–30 s, and 4–9%, 0.6–1.2%, and 0.25–0.38% as double-, triple-, and fully labeled forms, respectively. For aspartate, 66–68%, 5–8%, 0.5–1.1%, and 0.22–0.37% was present as single-, double-, triple-, and fully labeled isotopomers, respectively. Labeling in PEP was low for the first 60 s and then rose (Fig. 1). Subsequently, intermediate-labeled forms of malate^a^ and aspartate increased and declined, with the fully labeled form being dominant from 10 min onwards. This was accompanied by appearance of label in pyruvate and alanine (Fig. 1).

To estimate position-dependent enrichment in the C_4_ position and the C1–C3 positions of malate and aspartate, we assumed all labeled molecules contained ^13^C at the C_4_ position (see Supplementary Table S7 for the calculation). This assumption is justified by the very low PEP labeling at early time points (Fig. 1; Supplementary Table S6). Estimated enrichment in the C_4_ position averaged 71% in the first 10–30 s, and approached 100% at 40–60 min. Enrichment in C1–C3 was negligible at early times, and from 30 s on rose gradually to >80% after 60 min (Supplementary Table S7).

### Enrichment kinetics after correcting for inactive pools and pathway topology

**Fig. 2.**
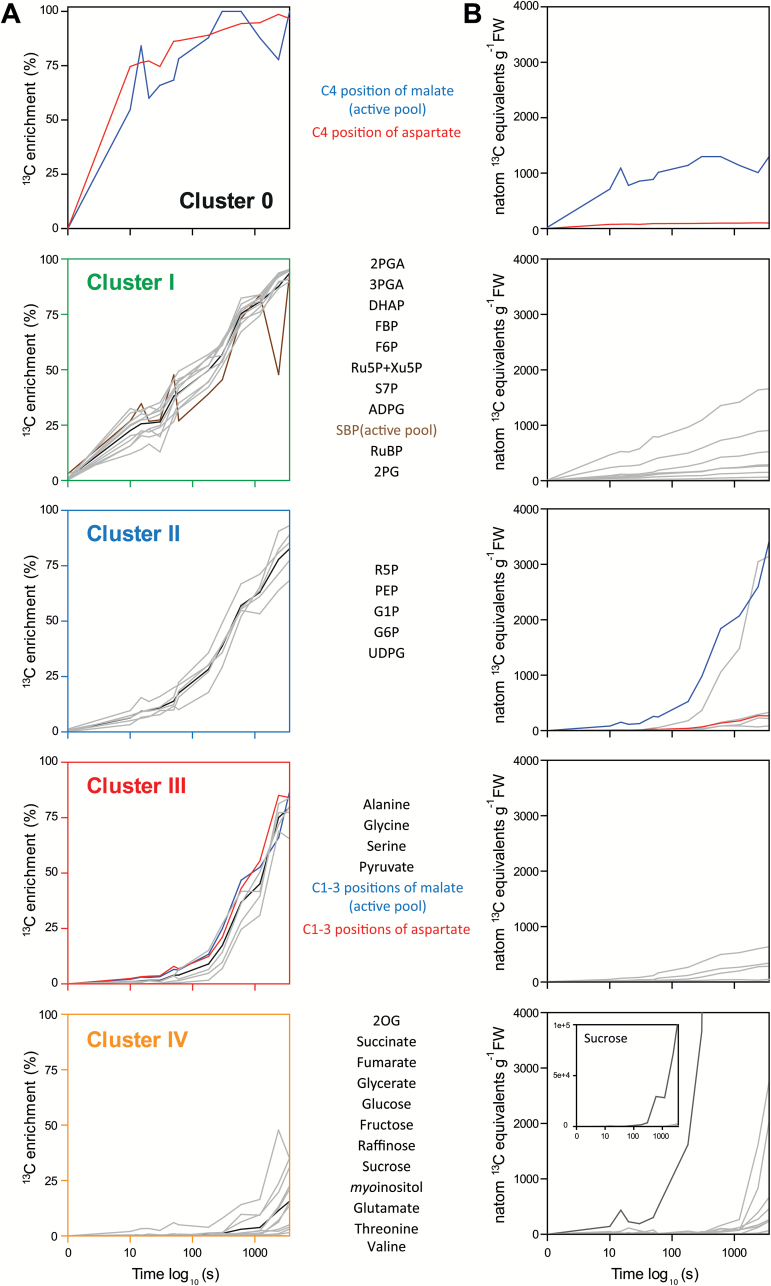
Overview of ^13^C labeling kinetics by *k*-means clustering (A) and corresponding ^13^C amounts (B) after correction for inactive pools and for labeling of malate and aspartate in the C4 positon and the C1–C3 positions. Gray lines show the ^13^C enrichment/^13^C amount (in natom ^13^C equivalents g^−1^ FW) of individual metabolites, and black lines show average ^13^C enrichment of all metabolites in the cluster. The *x*-axis corresponds to the labeling time on a log_10_ scale. Carbon position-dependent enrichments and ^13^C amount were separately calculated for the C4 position and C1–C3 positions of malate and aspartate (for further information about calculations, see Supplementary Tables S7 and S8). Data for other ^13^C enrichments and ^13^C amounts are provided in Supplementary Table S5 and S8 (with further information about calculations). The corrected ^13^C enrichment for SBP is shown in brown, and for malate and aspartate in blue and red, respectively. In (B), metabolites are clustered based on enrichment, not the amount of ^13^C in the metabolite. Due to a large amount of ^13^C in sucrose (B, last graph, shown in darker gray), an additional graph for this metabolite is included as an insert.

In Fig. 2A, adjusted values for ^13^C enrichment in malate, aspartate, and SBP, after correction for inactive pools for malate and SBP and separation of the C_4_ and C1–C3 positions of malate and aspartate, are superimposed on the *k*-means clusters in Supplementary Fig. S4. The C_4_ positions of malate and aspartate are shown as cluster 0 whose enrichment rises more rapidly than the CBC intermediates in cluster I. PEP labels more slowly than CBC intermediates and is in cluster II. Enrichment in the C1–C3 positions of malate and aspartate rises even more slowly, resembling pyruvate and alanine, which are in cluster III.

The similarity of its enrichment kinetics to those of malate^a^ indicates that aspartate contributes to the CCS. Further evidence was provided by the chase experiment in which leaves were labeled for 60 min and chased with ^12^CO_2_ for 5 min and 20 min. Label depleted more rapidly in aspartate than malate, even after correction for the inactive malate pool (Supplementary Fig. S7).

The rapid labeling of CBC metabolites, with multiple labeled isotopomers appearing within 10 s (Supplementary Fig. S5), resembles the pattern in tobacco and Arabidopsis (Hasunuma *et al.*, 2009; Szecowka *et al.*, 2013; Ma *et al.*, 2014). After 40–60 min, 67–81% of CBC intermediates were present as fully labeled isotopomers. Incomplete labeling at the end of the kinetics was also reported in tobacco and Arabidopsis (Hasunuma *et al.*, 2009; Szecowka *et al.*, 2013; Ma *et al.*, 2014).

Starch is synthesized from F6P via G6P, G1P, and ADPG in BSC chloroplasts, and sucrose is synthesized from triose-P via FBP, F6P, G6P, G1P, and UDPG in the MC cytosol (Furbank *et al.*, 1985; Hatch and Osmond, 1976; Stitt and Heldt, 1985*a*). ADPG had enrichment kinetics similar to the CBC intermediates (Fig. 2A). This is explained because the small pool (0.7 nmol g^−1^ FW) will turn over rapidly, as in Arabidopsis (Szecowka *et al.*, 2013). G6P, G1P, and UDPG were labeled more slowly than the CBC intermediates (Fig. 2A; Supplementary Fig. S5). F6P was labeled more rapidly than G6P, reflecting its additional role in BSCs as a CBC intermediate. Compared with G6P, labeling of UDPG and G1P was slightly and markedly slower. The proportion of unlabeled form at the end of the experiment was negligible for F6P (4.2%) and G6P (6.8%), and substantial for UDPG (17%) and G1P (30%), while the rest was predominantly fully labeled isotopomers. This resembles the pattern in Arabidopsis and tobacco (Hasunuma *et al.*, 2009; Szecowka *et al.*, 2013) and indicates that some of the UDPG and much of the G1P are not involved in photosynthesis.

### Calculation of the amount of ^13^C in metabolites

We combined information about metabolite content, isotopomer distribution, and the number of ^13^C atoms in an isotopomer to calculate the amount of ^13^C in each metabolite at a given time (Fig. 2B). In view of the variation in pool size, we compared two approaches: multiplying isotopomer distribution at a given time point by the average total pool size (or active pool size determined at 60 min), or using the sum of the measured isotopomer amounts at that time point (see Supplementary Table S8 for further explanation). The relatively good agreement between the approaches provided support for their reliability (shown for malate and aspartate in Supplementary Fig. S8). All ^13^C amounts presented in this study were calculated using the first approach.

The estimated ^13^C pool in the C_4_ position of malate and aspartate, respectively, rose from ~700 nmol and 80 nmol ^13^C equivalents g^−1^ FW at 10 s to 1300 nmol and 100 nmol ^13^C equivalents g^−1^ FW from 60 s onwards (Fig. 2B; Supplementary Tables S7, S8). The higher value for malate reflects the 10-fold larger pool size of active malate compared with aspartate (Supplementary Table S4). Comparison of the amount of ^13^C in the C_4_ position of malate and aspartate in the initial 20 s of the labeling kinetics (Table 1) indicates that ~10-fold more carbon is shuttled into the BSCs via malate than via aspartate.

**Table 1.** Estimations of ^13^C fluxes via malate and aspartate to the BSCs Amounts of ^13^C are expressed as natom ^13^C equivalents g^−1^ FW. Calculation steps are presented in Supplementary Tables S7 and S8.

	Kinetics (s)		
	10	15	20
Malate	792	1242	893
Malate minus C4 position	81	150	113
C en route to BSCs via malate (C4 position)	711	1092	780
Aspartate	85	89	91
Aspartate minus C4 position	7.5	9.9	10.8
C en route to BSCs via aspartate (C4 position)	78	80	80
**Aspartate/malate ratio**	**0.11**	**0.073**	**0.10**

The amount of ^13^C in the C_4_ position of malate and aspartate is large compared with the total pool of CBC intermediates (Fig. 2B). From 1 min on, it is 30- to 50-fold larger than the pool of RuBP (32 ± 11 nmol g^−1^ FW) and only ~3-fold smaller than the total carbon in CBC intermediates (~4100 nmol C g^−1^ FW). The amount of ^13^C in alanine is nearly 10-fold higher than that in pyruvate. It is also noteworthy that from 180 s onwards, the vast majority of the detected ^13^C is in sucrose (note insert in Fig. 2B). Negligible amounts of ^13^C were found in fumarate, succinate, 2OG, and glutamate up to 20 min and, even after 60 min, they contained only 0.01, 0.18, 0.52, and 2.2%, respectively, of the total detected label compared with 83.7% in sucrose. Although the ^13^C enrichment kinetics of sucrose and these organic acids and amino acids are similar (Fig. 2A), there is much more ^13^C in sucrose because its pool is much larger (Supplementary Table S4).

### Carbon exchange between the CBC and CO_2_ concentration shuttle intermediates

At early time points. enrichment is higher in PEP than in pyruvate (Supplementary Table S5; Fig. 1) indicating that PEP is probably labeled from the CBC. Carbon exchange between the CBC and CCS will occur via interconversion of PEPC, 3PGA, 2PGA, and PEP via the reversible reactions catalyzed by phosphoglycerate mutase and enolase (Newsholme and Start, 1973). Overall enrichment in 3PGA and 2PGA was almost 10-fold higher than in PEP at early time points (10–30 s) and was still 3-fold higher after 3 min (Fig. 1; Supplementary Table S5), implying some kinetic restriction on flow of newly assimilated ^13^C.

To estimate flow of ^13^C from the CBC into the metabolites of the CCS, we summed the amount of ^13^C in PEP, in C1–C3 of malate and aspartate, and in pyruvate and alanine (i.e. ^13^C atoms in CCS intermediates excluding the C_4_ position of malate and aspartate; Table 2). It represented 8.7–13.1% of total detected ^13^C at the initial time points (10, 15, and 20 s).

**Table 2.** Estimations of ^13^C fluxes of C from the CBC into CO_2_ shuttle intermediates Amounts of ^13^C are expressed as natom ^13^C equivalents g^−1^ FW. Calculation steps are presented in Supplementary Table S8.

Class of metabolite	Kinetics (s)		
	10	15	20
CO_2_ shuttle minus C4 position for malate and aspartate	103	186	155
CBC	1003	1131	1134
Starch and sucrose synthesis	72.2	99.4	105.9
Photorespiration	2.9	1.3	2.9
Total C fixed via CBC	1187	1424	1404
**% of fixed** ^**13**^ **C found in CO**_**2**_ **shuttle metabolites**	**8.7**	**13.1**	**11.1**

### Labeling kinetics of photorespiratory intermediates

We found substantial label in photorespiratory metabolites. At 10–30 s, average enrichment in 2PG was 14% (Supplementary Table S5), compared with 29% and 30% in RuBP and 3PGA. Sequential increase of enrichment in 2PG, glycine, serine, and glycerate (Fig. 2A; Supplementary Fig. S9) is explained by these metabolites being in a linear pathway, with the latter three having large active pools (1.7, 84, 166, and 425 nmol g^−1^ FW for 2PG, glycine, serine, and glycerate, respectively). Movement through glycolate and glyoxylate, which was not measured, may lead to a further delay. Unlabeled isotopomer forms for glycine and serine plateaued between 40 min and 60 min, suggesting the presence of inactive pools (Supplementary Fig. S9; ~22% and 20%, respectively). This was observed for glycine but not serine in Arabidopsis (Szecowka *et al.*, 2013).

We did not estimate C flow through photorespiration from the early time points of the labeling kinetics because the 2PG pool is very small and label will have moved more into glycolate and glyoxalate, which were not detected. Also GC-MS detects only incomplete fragments of glycine, serine, and glycerate. Gas exchange measurements in ambient CO_2_ revealed a 5.3 ± 1.7% stimulation of net photosynthesis after decreasing O_*2*_ from 21% to 2% (Supplementary Table S9).

### Comparison of maize with the C_3_ plant Arabidopsis in ambient and low O_2_

**Fig. 3.**
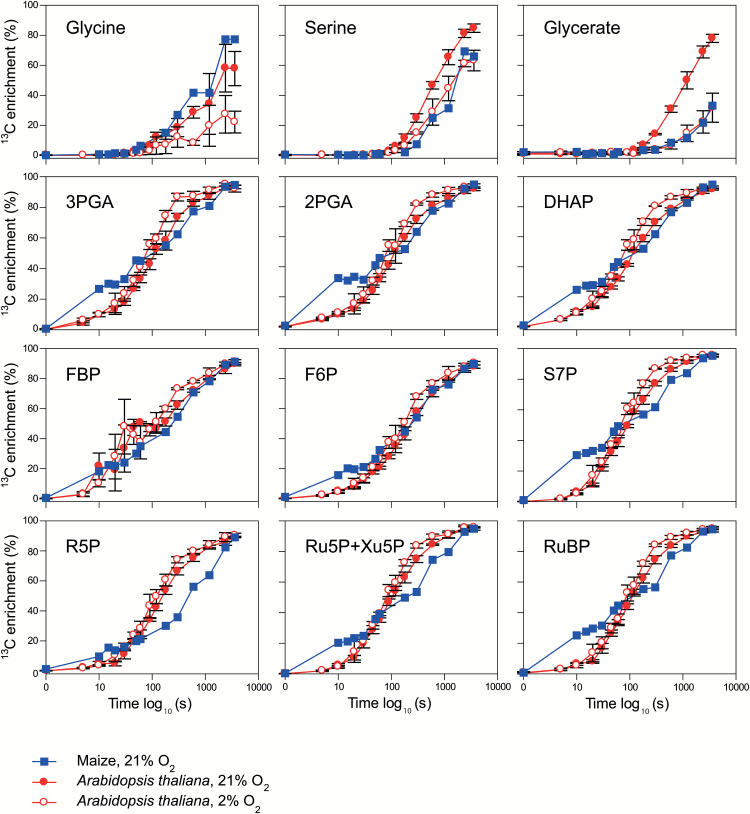
Comparison of ^13^C enrichment (%) of photorespiratory pathway and CBC intermediates in maize at ambient O_2_ and *Arabidopsis thaliana* at ambient and low O_2_. The *x*-axis corresponds to the labeling time on a log_10_ scale. Values are means ±SD (*n*=3–13). Data are presented in Supplementary Tables S5 and S10.

We next compared labeling kinetics in maize and Arabidopsis. To distinguish between differences that are related to and independent of photorespiration, we labeled Arabidopsis at 420 ppm CO_2_ with either 21% or 2% O_2_. Enrichment of glycine, serine, and glycerate in Arabidopsis in 21% O_2_ (Fig. 3, red filled circles) resembled that in Szecowka *et al.* (2013). The slightly lower enrichment of glycine compared with serine and glycerate is explained by an inactive pool of glycine (Supplementary Fig. S11; Supplementary Table S10; see also Szecowka *et al.*, 2013). In 2% O_2_ (Fig. 3, red open circles), enrichment in photorespiratory intermediates was lower and rose more slowly. There were also significantly lower contents of serine and glycerate in 2% than in 21% O_2_ (Supplementary Fig. S10). Enrichment of CBC intermediates increased more rapidly in 2% than 21% O_2_, especially between 45 s and 10 min. This is probably due to faster ^13^C assimilation and decreased recycling of ^12^C from photorespiratory metabolites into the CBC.

In maize (Fig. 3, blue filled squares), the enrichment patterns for serine and glycerate resembled those in Arabidopsis in 2% O_2_. Enrichment of glycine was higher than in Arabidopsis, but interpretation is complicated by the inactive pool. As expected from the higher rate of photosynthesis, enrichment in CBC metabolites in the first 3 min was higher in maize (Fig. 3, blue filled squares) than in Arabidopsis, even in 2% O_2_. However, between 5 min and 20 min, enrichment of CBC metabolites was similar or slightly lower in maize than in Arabidopsis in 21% O_2_, and was always lower than in Arabidopsis in 2% O_2_. By 40–60 min, enrichment in maize was similar to that in Arabidopsis. This labeling pattern indicates that in maize between 5 min and 20 min, unlabeled C flows into the CBC that probably does not derive from photorespiration.

### Fractionation to enrich MCs and BSCs

Maize leaf material was fractionated to enrich MCs and BSCs partially in order to estimate intercellular concentration gradients for CCS metabolites, 3PGA and triose-P, and to provide independent evidence for parallel operation of different CCSs. It is difficult from labeling kinetics, on their own, to discriminate if labeling of aspartate and alanine is due to their synthesis in one cell type and consumption in the other cell type (i.e. involvement in the CCS) or to reversible formation by aminotransferase reactions in one or both cell types without them being involved in net flux. The presence of a concentration gradient between BSCs and MCs would provide independent evidence for their involvement.

Fractions enriched in MCs and BSCs were obtained by sequentially filtering leaf homogenates in liquid N_2_ through three nylon meshes with different pore sizes (Stitt and Heldt, 1985*a*). BSCs are mechanically stronger and are enriched in larger particles that are retained on meshes with a larger pore size, whereas MC-enriched material is preferentially retained on smaller meshes or passes through to the final filtrate. The leaf discs obtained with the labeling chamber did not provide enough tissue for this procedure. Instead, leaves were incubated in transparent plastic bags (see Supplementary Fig. S2E, G, H and the Materials and Methods for details). A 3 min pulse was used, when all isotopomers of malate, aspartate, pyruvate, alanine, and PEP are at high enough levels to be reliably quantified.

We first compared metabolite amounts and labeling patterns in non-filtered homogenates with those at 3 min in the kinetic experiment. Metabolite levels were similar in both experiments (Supplementary Table S4), with a few exceptions; in material for cell separation, aspartate was 3- to 4-fold higher, R5P, Ru5P+Xu5P, and RuBP were slightly higher, glucose 2-fold lower, and sucrose 7-fold lower. This may be partly because the tissue collected for cell separation included a larger part of the leaf than the leaf disc harvested with the labeling chamber. As previously mentioned, there are substantial metabolic gradients along the maize leaf blade (Pick *et al.*, 2011; Wang *et al.*, 2014*a*).

^13^C Enrichment in the various metabolites was higher at 3 min in the tissue fractionation experiment than at 3 min in the kinetic experiment (Supplementary Table S5). Correlation analysis revealed that ^13^C enrichment values in the tissue fractionation experiment resembled those after 5 min in the time kinetic experiment (Supplementary Fig. S12). This indicates that there were higher rates of photosynthesis in the tissue fractionation experiment. All key features of the time kinetic experiment were confirmed in the triplicated samples for tissue fractionation (Supplementary Table S5). All CBC metabolites were highly labeled (53 ± 5% to 69 ± 3% ^13^C enrichment) with the exception of SBP (9 ± 6% enrichment when not corrected for the inactive pool), ADPG was strongly labeled (63 ± 2%), malate and aspartate were present mainly in the single-labeled form (45 ± 10% for malate after correction, 52 ± 3.5% for aspartate), PEP was less enriched than 2PGA and 3PGA (30 ± 2% compared with 69 ± 3 and 67 ± 0.67%, respectively), pyruvate and alanine were relatively weakly labeled (18 ± 1% and 14 ± 2% enrichment, respectively, there was substantial labeling of glycine and progressively less of serine and glycerate (36 ± 2, 11 ± 11, and 1.9 ± 0.58%, respectively; 2PG was not analyzed in this experiment), and there was negligible labeling of succinate, 2OG, and glutamate (0.09 ± 0.16, 0.66 ± 0.27, and 0.60 ± 0.2% enrichment, respectively).

### Intercellular metabolite distributions and metabolite gradients in maize leaves

**Fig. 4.**
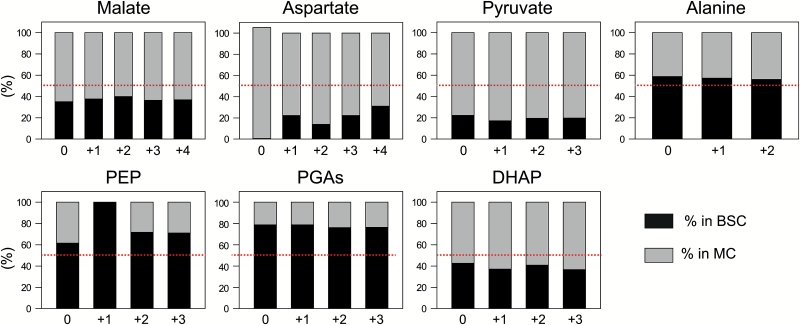
Distribution of isotopomers between BSCs and MCs. Labeling was performed for 3 min. The number associated with the compounds represents the number of ^13^C incorporated in the molecule. The *y*-axis shows the percentage of that isotopomer in the BSCs (black) and MCs (gray). The dotted red line indicates 50%. Data are provided in Supplementary Table S12.

We used PEPC and NADP-MDH activities as markers for the MCs and total RuBP as a marker for the BSCs. They were compared with isotopomer amounts in the four fractions from the net filtration (Supplementary Table S11) to estimate the distribution of each isotopomer of the other metabolites between the BSCs and MCs (Fig. 4; Supplementary Table S12). As the signals for 3PGA and 2PGA could not be differentiated in all fractions, they were combined and termed ‘PGAs’.

As shown in Fig. 4, malate and aspartate were preferentially located in the MCs, with aspartate showing an especially marked asymmetric distribution. Alanine showed a slight preferential distribution towards the BSCs, pyruvate was preferentially located in the BSCs, PEP showed a strong preferential distribution towards the BSCs, PGAs was preferentially located in the BSCs, and triose-P in the MCs. In all cases, a similar distribution was found for the unlabeled form and each isotopomer.

The amount in the BSCs and MCs (Table 3) was estimated by multiplying the fraction of the summed isotopomers found in the BSCs and the MCs, respectively, by the average active pool size from the time kinetic/chase experiment (Supplementary Table S4). For malate, the fraction of summed ^13^C isotopomers was multiplied by the active malate pool size at 60 min (Supplementary Table S3). We then related these amounts to the cytoplasmic volume since diffusion will occur between the cytoplasm of the BSCs and the MCs. For this, we assumed that BSCs occupy 19% of the leaf (Furbank and Hatch, 1987; Jenkins *et al.*, 1989), that MCs have an ~3.8-fold larger volume than the BSCs (Hattersley, 1984), and that the cytoplasm occupies ~50% of BSC volume (Furbank and Hatch, 1987; Jenkins *et al.*, 1989) and 10% of MC volume (Osmond, 1971). These published data allow us to take into account that the BSCs and MCs occupy a different proportion of the maize leaf, and have a different internal architecture.

**Table 3.** Estimations of overall metabolite concentrations (mM) involved in photosynthesis in BSCs and MCs of maize leaves Concentrations are calculated from the distribution of summed isotopomers (Supplementary Table S12) and metabolite amounts quantified in non-filtrated homogenates (Supplementary Table S4). The difference between concentrations in MCs and BSCs gives the concentration gradient between these two cell types. Due to the presence of an inactive malate pool, the unlabeled isotopomer amount was not included for calculation and the total active pool estimated at 60 min was used (L stands for labeled pool).

	mM		
Compound	BSCs	MCs	Concentration gradient (MCs to BSCs)
Malate(L)	5.20	11.18	5.99
Aspartate	0.21	1.17	0.96
Pyruvate	0.26	1.53	1.27
Alanine	11.33	10.65	–0.68
PEP	0.79	0.38	–0.40
PGAs	4.73	1.87	–2.87
DHAP	1.29	2.73	1.43

We estimated a malate concentration of 11.2 mM in the MCs and 5.2 mM in the BSCs, resulting in a large (6 mM) gradient between MCs and BSCs (Table 3). Aspartate was even more asymmetrically distributed, with estimated concentrations of 1.17 mM in the MCs and 0.21 mM in the BSCs, but the smaller pool size means that the concentration gradient (0.96 mM) was about a sixth of that for malate. These concentration gradients are in the direction needed to drive diffusion of malate and aspartate in the BSCs. Pyruvate showed a very asymmetric distribution with estimated concentrations of 1.53 mM in the MCs and 0.26 mM in the BSCs, resulting in a concentration gradient of 1.27 mM in the opposite direction to that required to drive diffusion of pyruvate from the BSCs to the MCs. Alanine had an estimated concentration of 11.5 mM in the BSCs and 10.8 mM in the MCs, leading to a small concentration gradient (0.68 mM) from the BSCs to the MCs. Alanine showed only a weakly asymmetric distribution between the MCs and BSCs, making the calculation sensitive to errors in the assumed volumes. 3PGA had a higher estimated concentration in the BSCs (4.73 mM) than in the MCs (1.87 mM), and triose-P had a higher estimated concentration in the MCs (2.73 mM) than in the BSCs (1.29 mM), providing a concentration gradient of 2.87 mM to drive diffusion of 3PGA from the BSCs to the MCs, and 1.43 mM to drive diffusion of triose-P from the MCs to the BSCs. PEP concentrations of 0.79 mM and 0.30 mM were estimated for the BSCs and MCs, respectively, providing a small (0.4 mM) concentration gradient from the BSCs to the MCs. Although the absolute magnitude is small, it is based on a highly asymmetric distribution of PEP. The PEP:3PGA ratio in the MCs (0.166) and BSCs (0.2) resemble those expected if 3PGA and PEP are close to thermodynamic equilibrium (Newsholme and Start, 1973).

## Discussion

### Overview of temporal labeling kinetics in C_4_ photosynthesis in maize

Some of our results recapitulate the studies with ^14^CO_2_ that led to the elucidation of C_4_ photosynthesis, in particular the rapid labeling of C4 acids and slower labeling of pyruvate (Slack and Hatch, 1967; Hatch, 1971; Hatch and Osmond, 1976). A recent study of the role of the DCT2 transporter reported rapid labeling of malate and aspartate, and labeling of photorespiration intermediates (Weissmann *et al.*, 2016). However, we have analyzed a larger set of metabolites and included longer labeling kinetics to allow separation of active and inactive pools. Further, we have determined metabolite concentrations and labeling patterns in the MCs and BSCs, and estimated concentration gradients between the MCs and BSCs. Our analyses provide information about which metabolites are involved in the CCS and how rapidly carbon is exchanged between the CBC and CCS, and raise questions about how metabolism is regulated to allow efficient operation of C_4_ photosynthesis.

Our approach has some limitations. First, it is not possible to resolve CBC labeling kinetics (see also Szecowka *et al.*, 2013; Ma *et al.*, 2014). However, rapid randomization aids interpretation of labeling kinetics in metabolites downstream of the CBC. Secondly, overall metabolite levels show leaf-to-leaf and within-leaf variation in maize (Leegood and von Caemmerer, 1988; Pick *et al.*, 2011; Wang *et al.*, 2014*a*). The labeled pools showed less variation, indicating that the variation is mainly due to pools not directly involved in photosynthesis. Thirdly, some metabolites exhibit complex labeling kinetics, with part remaining unlabeled while the remainder is present as multiple or fully labeled isotopomers. We interpret this as evidence for the presence of multiple pools, with only one being active in photosynthesis (Szecowka *et al.*, 2013; Ma *et al.*, 2014). Separation of active and inactive pools is essential to estimate concentrations and fluxes, and can be achieved by labeling for long enough to label the active pool fully. Fourthly, as MS does not provide position-specific information, we distinguished between label in the C_4_ and the C1–C3 positions of malate and aspartate by assuming all labeled forms have a ^13^C at the C_4_ position. This approach is supported by the similarity between the labeling kinetics we estimated for positions C1–C3 of malate and aspartate, and measured for pyruvate and alanine. Fifthly, we only obtain partial enrichment of MCs and BSCs, and errors are introduced in corrections for cross-contamination. In addition, our calculations of concentrations use published values for cell and cytoplasmic volume (Osmond, 1971; Hattersley, 1984; Furbank and Hatch, 1987; Jenkins *et al.*, 1989). For these reasons, the estimated MC and BSC concentrations are only approximations.

An ultimate goal is to use global enrichment kinetics to model fluxes. However, this is complicated by redundancy in C_4_ photosynthesis. Based on labeling kinetics alone, it is difficult to decide whether ^13^C is detected in aspartate and alanine because they are directly involved in C_4_ photosynthesis or because they exchange ^13^C with OAA and pyruvate. A similar problem arises for formation of PEP in the BSCs, which may be labeled by decarboxylation of labeled four-carbon metabolites or ^13^C exchange with 3PGA. For this initial study, we restricted our calculations to early points in the time kinetics, and tried to support these calculations by estimating intercellular concentration gradients to provide independent evidence that flux is carried by a particular metabolite.

### Concentration gradients to drive intercellular metabolic shuttles

Earlier studies reported gradients for 3PGA movement from the BSCs to the MCs, and for malate and triose-P movement from the MCs to the BSCs, but no gradient for pyruvate (Leegood, 1985; Stitt and Heldt, 1985*a*, *b*). These studies assumed the whole pool was involved in C_4_ photosynthesis, as opposed to being partly located in, for example, the vacuole or other cell types. The observation that large amounts of malate remain in darkness or at CO_2_ subcompensation point indicated that this might be the case (Stitt and Heldt, 1985*b*). Similarly, it remained possible that compartmentation masked a gradient for pyruvate. Dynamic ^13^CO_2_ labeling allows us to measure the size and distribution of the pools that are actually involved in photosynthesis, which we term the active pool. For malate, this represents ~40% of the total pool. Labeling of the other metabolites involved in the intercellular shuttles was high by 40–60 min, showing that most of their pool is involved in C_4_ photosynthesis.

The estimated concentration of the active malate pool is ~2-fold higher in the MCs than in the BSCs, resulting in an estimated gradient of ~6 mM from the MCs to the BSCs. 3PGA is preferentially located in the BSCs and triose-P in the MCs, providing estimated gradients of ~2.9 mM for 3PGA from the BSCs to the MCs, and 1.4 mM for triose-P from the MCs to the BSCs. There were also concentration gradients for further metabolites including a gradient for PEP from the BSCs to the MCs (0.4 mM), for aspartate from the MCs to the BSCs (0.96 mM), and for alanine from the BSCs to the MCs (0.68 mM) (see below for more discussion).

The labeled pyruvate pool showed strong preferential distribution towards the MCs. It therefore remains unclear how pyruvate moves from the BSCs to the MCs. One possibility is that after its formation in the BSC chloroplasts, pyruvate is actively exported to the cytosol, or that pyruvate is actively taken up into MC chloroplasts. Active pyruvate uptake has been measured in MC chloroplasts (Flügge *et al.*, 1985). While a sodium-dependent transporter has been identified in *Flaveria* species (Furumoto *et al.*, 2011), pyruvate transporters have not yet been identified in species such as maize where uptake is proton dependent (Aoki *et al.*, 1992). The transporter that exports pyruvate from BSC chloroplasts is also unknown.

The estimated gradients for malate, 3PGA, and DHAP in our study are smaller than those previously reported for overall pools (Leegood, 1985; Stitt and Heldt, 1985*a*, *b*). This is probably because we used a lower irradiance (480 µmol photons m^−2^ s^−1^ compared with 1300–2500 µmol photons m^−2^ s^−1^). It should be noted that diffusion will be driven by the concentration gradient between the BSC and MC cytosols. Our calculations assume an equal distribution of metabolites between the cytoplasm and chloroplast, and the gradients will be different if the metabolite is preferentially located in one of these compartments. This may explain why the estimated concentration gradient is larger for 3PGA than for triose-P. Due to the pH gradient between the plastid stroma and the cytosol and their charge properties, 3PGA is preferentially located in the stroma and triose-P in the cytosol (Stitt *et al.*, 1980; Flügge *et al.*, 1983; Gerhardt and Heldt, 1984).

### Involvement of aspartate and alanine in the CO_2_-concentrating shuttle

It has been proposed that NADP-ME-type plants such as maize operate parallel CCSs via PEPCK or NAD-ME (Pick *et al.*, 2011). Aspartate aminotransferase (AspAT) activity is high in maize leaves (Khamis *et al.*, 1992; Pick *et al.*, 2011; Wang *et al.*, 2014*a*), alanine aminotransferase (AlaAT) activity is only 3- to 10-fold lower (Pick *et al.*, 2011; Valle and Heldt, 1991; Wang *et al.*, 2014*a*), and PEPC, AspAT, and AlaAT show very similar developmental profiles (Pick *et al.*, 2011; Wang *et al.*, 2014*a*; data replotted in their Supplementary Fig. S3). PEPCK is expressed in the BSCs and supports high rates of aspartate-dependent photosynthesis in isolated maize BSCs (Walker *et al.*, 1997; Wingler *et al.*, 1999; Majeran *et al.*, 2010; Pick *et al.*, 2011). Many studies reported the presence of substantial pools of aspartate and rapid labeling of aspartate in NADP-ME species such as maize (Hatch, 1971; Meister *et al.*, 1996; Wingler *et al.*, 1999; Majeran *et al.*, 2010; Pick *et al.*, 2011; Sommer *et al.*, 2012; Muhaidat and McKown, 2013; Weissmann *et al.*, 2016). However, these studies remained inconclusive because it was unclear how strongly aspartate was labeled and whether there was a concentration gradient to drive its movement from the MCs to BSCs.

Our study reveals that there is a rapid and near complete labeling of aspartate, that the enrichment kinetics and isotopomer distribution of aspartate are almost identical to those of the active malate pool, and that label in aspartate is rapidly lost in a chase. They also reveal a strongly asymmetric distribution of aspartate, resulting in a distinct concentration gradient between the MCs and BSCs. The gradient (0.96 mM) is ~6-fold smaller than the estimated gradient for malate, consistent with a shuttle involving aspartate carrying ~4% of the CO_2_ into the BSCs. This is in quite good agreement with the initial labeling kinetics estimated for the C_4_ positions of malate and aspartate, which indicate that aspartate carries ~10% of the carbon into the BSCs. Our estimates are lower than the 34% estimated by Weissmann *et al.* (2016) (see below for further discussion).

In the BSCs, aspartate might be deaminated and decarboxylated by PEPCK, or further reduced to malate and decarboxylated by NAD-ME or NADP-ME (Furbank, 2011; Pick *et al.*, 2011; Wang *et al.*, 2014*b*). In the former case, PEP is formed and would have to return to the MCs. Both the overall and the labeled pool of PEP are ~4-fold higher in the BSCs than in the MCs, providing a concentration gradient of ~0.4 mM to drive movement from the BSCs to the MCs. If aspartate moves from the MCs to the BSCs, amino groups will have to return to the MCs. Weissmann *et al.* (2016) postulated that they might return as alanine. Our study reveals that the labeling kinetics of alanine are almost identical to those of pyruvate, and that alanine is strongly enriched by 40–60 min, implying that most of the large alanine pool is involved in C_4_ photosynthesis. Further, and in contrast to pyruvate, we estimate a small concentration gradient for alanine (0.69 mM) between the BSCs and MCs. It should, however, be noted that the alanine pool showed only a slight asymmetric distribution, so this estimate will be sensitive to noise in our data and our assumptions.

Pick *et al.* (2011) proposed that the NADP-ME species maize operates a parallel CCS via PEPCK. This proposal was based on PEPCK showing a similar developmental gradient to other C_4_ enzymes and having a high expression and activity in the BSCs. Our study supports this idea. The estimated concentration gradient for movement of aspartate to the BSCs was 0.96 mM, compared with gradients of 0.4 mM and 0.68 mM for movement of PEP and alanine back to the MCs. Making the reasonable assumption that fluxes are proportional to the intercellular concentration gradients, ~40% of the aspartate may be decarboxylated by PEPCK and the remainder by NAD- or NADP-ME. However, it is important to stress that these estimates are approximate. It is also possible that some aspartate is converted to malate in the BSCs and decarboxylated by NADP-ME. This pathway would not transfer reducing equivalents from the MCs to the BSCs. It operates in some NADP-ME species such as *Flavaria bidentis*, which have high PSII activity in the BSCs (Meister *et al.*, 1996). In maize, it is presumably restricted by low BSC PSII, but a minor contribution might be possible especially if, as postulated by Meister *et al.* (1996), PSII activity were to co-vary with aspartate decarboxylation via NADP-ME. There is some evidence that the distribution of PSII between the MCs and BSCs in maize can vary (Drozak and Romanowska, 2006).

It has been proposed that operation of multiple decarboxylation pathways is advantageous because it allows several metabolites to be involved in the CCS, permitting a given rate of photosynthesis to be achieved with lower concentrations of each individual metabolite (Wang *et al.*, 2014*b*). It may also confer robustness in a fluctuating environment (Furbank, 2011; Stitt and Zhu, 2014; Wang *et al.*, 2014*b*). There may be considerable flexibility in the extent to which different decarboxylation pathways are used, even in a given species. While there was a marked asymmetric distribution of aspartate between the MCs and BSCs, and a gradient of PEP between the BSCs and MCs in the present study, gradients for aspartate (Leegood, 1985; Stitt and Heldt, 1985*a*, *b*) and alanine (Leegood, 1985) were not detected previously, and gradients for PEP were either not detected (Stitt and Heldt, 1985*a*, *b*) or were in the opposite direction (Leegood, 1985). The overall aspartate:malate ratio varies between studies, with reported values ≤0.1 (Leegood and Furbank, 1984; Leegood, 1985), as high as 0.2 (Khamis *et al.*, 1992; Usuda, 1985), or even 0.5 (Usuda, 1987; Leegood and von Caemmerer, 1988; Pick *et al.*, 2011), and <0.1 in our kinetic experiment and >0.2 in the material used to analyze intercellular distributions. While alanine levels are usually about half the overall malate content, this can vary (Leegood, 1985; Pick *et al.*, 2011), and the relative levels of aspartate, alanine, and malate vary between individual leaves (Leegood and von Caemmerer, 1988) and within a single leaf (Wang *et al.*, 2014*a*). Further experiments are needed to understand the reason for this variation, which may reflect differing contributions of NADP-ME and other decarboxylation enzymes, and its consequences for C_4_ photosynthesis under different environmental conditions.

Operation of the CCS requires that the high concentrations of organic acids and amino acids are insulated from wasteful respiration (Bräutigam *et al.*, 2014), including insulation. Our labeling kinetics show that whereas the active malate pool, aspartate, pyruvate, and alanine approach full enrichment by ~20 min, there was negligible movement of ^13^C into TCA cycle intermediates such as succinate or 2OG, and amino acids derived from these organic acids.

### Flux between the CBC and the CO_2_-concentrating shuttle

In maize, C_4_ photosynthesis requires two types of intercellular shuttle: the CCS that deliver CO_2_ to the BSCs and the 3PGA/triose-P shuttle that supplies NADPH and ATP to the BSCs. This raises the question of how they are co-ordinated. Excess CCS would result in CO_2_ overaccumulation in the BSCs and increased wasteful back leakage of CO_2_ to the MC. Up to 20–30% of the carbon incorporated by PEPC may leak back (Farquhar, 1983; Kromdijk *et al.*, 2014), which will decrease quantum yield because energy is required to drive the CCS. On the other hand, a low CCS would lead to a decrease in the BSC CO_2_ concentration and a wasteful increase in photorespiration (de Veau and Burris, 1989; von Caemmerer and Furbank, 2003; Bellasio and Griffiths, 2014). Flux in the shuttles will depend on the rate of diffusion, which depends on the magnitude of the concentration gradients and will therefore be constrained by total pool size. This makes it important to ask if carbon can be exchanged between the CCS and the 3PGA/triose-P shuttle and, if so, how quickly.

Earlier studies showed that there is a large increase in the levels of pyruvate, 3PGA, and DHAP but no marked change in the level of malate during induction of photosynthesis in maize (Leegood and Furbank, 1984; Usuda, 1985), and that higher light intensities lead to a progressive increase in the steady-state levels of pyruvate, 3PGA, and, especially, DHAP, but not of malate or PEP (Usuda, 1987; Leegood and von Caemmerer, 1988). It was proposed that carbon moves between the CCS and CBC, including the large pools in the 3PGA/triose-P shuttle (Leegood and Furbank, 1984; Usuda, 1985, 1987; Leegood and von Caemmerer, 1988). However, these conclusions were qualitative, and depended on comparisons at different light intensities or different times during the induction of photosynthesis.

^13^CO_2_ labeling allows carbon flow to be investigated during steady-state photosynthesis. Our study reveals considerable movement of carbon from the CBC into the CCS, with 9–13% of the ^13^C fixed by RuBisCO in the first 30 s moving into CCS metabolites. Further evidence for carbon exchange between the CBC and the CCS is provided by comparing the labeling kinetics of CBC intermediates in maize with the C_3_ plant Arabidopsis in 2% O_2_ where photorespiration is decreased to mimic the situation in maize. The initial increase of enrichment in CBC intermediates is faster in maize, as expected from its higher rate of photosynthesis. However, between 5 min and 20 min CBC metabolite enrichment increases more slowly in maize than in Arabidopsis, indicating that there is an influx of unlabeled carbon into the CBC in maize. The most likely source is the C1–C3 positions of malate and aspartate, and pyruvate and alanine. It requires >20 min for their enrichment to approach that of the CBC intermediates. This ^13^C is derived from the CBC (see above) and, as the experiments were carried out at metabolic steady state, there must be an equivalent flux of unlabeled carbon from malate, aspartate, pyruvate, and alanine into the CBC.

Taken together, our results reveal substantial movement of carbon between the CCS and the 3PGA/triose-P shuttle. During steady-state photosynthesis, this involves carbon exchange, but in non-steady-state conditions it will facilitate net carbon transfer. Total carbon in the CBC, including 3PGA and triose-P, was 4144 nmol g FW^−1^, total carbon in the CCS (positions C1–C3 of the active malate pool and aspartate, plus pyruvate and alanine) was ~11 000 nmol g FW^−1^, and the rate of photosynthesis was ~55 nmol CO_2_ g FW^−1^ s^−1^. Exchange between the CCS and CBC equivalent to 9–14% of the rate of photosynthesis could generate a 50% change in the total CBC pool including 3PGA and triose-P in ~10 min, and in the total pool of CCS metabolites in 30 min.

Our results highlight PEPC as important for regulating allocation between the CBC and CCS. The estimated concentration of PEP in the MCs (0.38 mM) is below the measured *K*_m_ of maize PEPC (0.6–1.5 mM; Ting and Osmond, 1973; Dong *et al.*, 1998) and especially the likely *K*_m_ in vivo in the presence of inhibitory metabolites and at low Mg^2+^ representative of that in the cytosol (Tovar-Mendez *et al.*, 2000). Changes in PEPC activity will alter the level of PEP and, via equilibration, 3PGA, as well as allocation between the CCS and CBC.

### Photorespiration

Our study and that of Weissmann *et al.* (2016) reveal a low but significant rate of photorespiration. We show that 2PG is labeled within 10 s, although less strongly than CBC intermediates. Label in 2PG represented <0.5% of fixed ^13^C at early time points, but the 2PG pool is very small and will turn over rapidly. The appearance of label in glycine, serine, and glycerate provides a better qualitative picture of the rate of photorespiration, and resembled the C_3_ plant Arabidopsis in 2% O_2_. Based on the response of photosynthesis in maize to O_2_, we estimate the rate of photorespiration in 420 ppm CO_2_ to be ~5% of that of carboxylation, which is ~5-fold lower than in a C_3_ plant in air (Sharkey, 1988; Florian *et al.*, 2014). However, this may depend on the conditions. For example, if PEPC activity were low, metabolite concentrations in the CCS would decrease (see above) with the risk that the supply of CO_2_ to the BSCs is decreased and photorespiration increased. This scenario might develop, for example, when stomatal closure leads to very low internal CO_2_ concentrations in the MCs. It might be speculated that this is one reason for the relatively high levels of photorespiratory enzymes in maize (Osmond and Harris, 1971; Ohnishi and Kanai, 1983; Ueno *et al.*, 2005) as in such conditions photorespiration might serve to increase energy dissipation.

## Supplementary data

Supplementary data are available at *JXB* online.

Fig. S1. Three major biochemical subtypes for C_4_ photosynthesis.

Fig. S2. ^13^C labeling systems and quenching procedures.

Fig. S3. Metabolic content of ^13^CO_2_-labeled maize leaf during the day.

Fig. S4. Overview of ^13^C labeling kinetics from primary carbon metabolism by *k*-means clustering.

Fig. S5. Time-course of mass distribution of metabolites from CBC, starch, and sucrose pathways.

Fig. S6. Scheme of positional carbon incorporation in compounds from the CO_2_ shuttle.

Fig. S7. Time-course of mass distribution of metabolites in malate and aspartate during a chase.

Fig. S8. Regression plots of ^13^C amounts calculated with two approaches.

Fig. S9. Time-course of mass distribution of photorespiration cycle intermediates, amino acids, organic acids, and sugars.

Fig. S10. Amounts of photorespiratory intermediates in *Arabidopsis thaliana* labeled with ^13^CO_2_ under 21% and 2% O_2_.

Fig. S11. Time-course of mass distribution of glycine in *Arabidopsis thaliana* labeled with ^13^CO_2_ under 21% and 2% O_2_.

Fig. S12. Regression plots of ^13^C enrichments from the kinetic experiment and material for cell separation.

Table S1. Specific isotopomer-dependent MS parameters used in selected reaction monitoring (SRM).

Table S2. Corconfig.txt file used with the CORRECTOR program for the correction of aspartate, PEP, 2PG, and pentose-phosphate data.

Table S3. Amounts of unlabeled form and [^13^C]isotopomers for each metabolite in the time kinetic pulse–chase experiments and the cell separation experiment.

Table S4. Metabolic content in the time kinetic pulse–chase experiments and the cell separation experiment.

Table S5. ^13^C enrichment (%) of metabolites in the time kinetic pulse–chase experiments and the cell separation experiment.

Table S6. Relative isotopomer abundance (%) in the time kinetic pulse–chase experiments and the cell separation experiment.

Table S7. Calculation of carbon-dependent ^13^C enrichment in the C_4_ position and in C1–C3 positions of malate and aspartate.

Table S8. Estimation of ^13^C amounts in the CO_2_ shuttle, CBC, and first intermediates of starch and sugar intermediates, photorespiratory intermediates, and additional metabolites

Table S9. Estimation of photorespiration in maize from gas exchange.

Table S10. *Arabidopsis thaliana* at ambient (21%) and low (2%) O_2_: ^13^C enrichment (%) of photorespiratory and CBC intermediates, relative isotopomer abundance (%) of glycine and serine, and amounts of photorespiratory intermediates.

Table S11. Metabolic content and activities of enzyme markers of the fraction obtained by cell separation of maize leaves.

Table S12. Distribution of isotopomers in BSCs and MCs of maize leaves.

## Supplementary Material

Supplementary_Figures_S1_S12

Supplementary_Tables_S1_S12
