# Supplementary material for: Metabolite pools and carbon flow during C4 photosynthesis in maize: 13CO2 labeling kinetics and cell type fractionation
Source: J Exp Bot. 2016 Nov 7;68(2):283–98. doi: 10.1093/jxb/erw414 (PMC5853532; doi:10.1093/jxb/erw414)

Supplementary Figure S1. Three major biochemical subtypes for C4 photosynthesis.

Pathways are indicated with black arrows, the major decarboxylation enzymes in bold blue and energy transfer in red. Chloroplasts and mitochondria are represented with green and purple borders, respectively. Adapted from Maier et al., (2011).

In the NADP-ME subtype **(A)**, OAA is reduced in MC chloroplasts by NADP-malate dehydrogenase (MDH) to malate, which moves to BSC chloroplasts where it is decarboxylated by NADP malic enzyme (NADP-ME), yielding CO_2, pyruvate and NADPH. Pyruvate returns to the MC chloroplasts and is phosphorylated by pyruvate phosphate dikinase (PPDK) to regenerate PEP. In the NAD-ME subtype **(B)**, OAA is converted in the MC by aspartate aminotransferase (AspAT) to aspartate, which moves to the BSC mitochondria where it is converted by AspAT to OAA, reduced by NAD-MDH to malate and decarboxylated by NAD-ME to yield CO_2, pyruvate and NADH. The NADH is consumed for OAA reduction. The pyruvate is converted by alanine aminotransferase (AlaAT) to alanine, which moves back to the MC, where it is deaminated by AlaAT to pyruvate that is used by PPDK to regenerate PEP. In the PEPCK subtype **(C)**, some of the OAA is converted to aspartate, which moves to the BSC cytosol, is deaminated to OAA and converted to CO_2 and PEP by PEPCK. PEP then returns to the MC. The remainder of the OAA is reduced to malate by NADP-MDH and moves to the BSC mitochondria where it is decarboxylated by NAD-ME, yielding CO_2, NADH and pyruvate. Pyruvate is converted to alanine which returns to MC and is deaminated to pyruvate, which is used to regenerate PEP. This NAD-ME and PEPCK variants additionally require intercellular movement of amino acids to maintain the NH_3 balance. In addition to CO_2, some of these shuttles transfer energy to the BSC; in the NADP-ME subtype NADPH is transferred allowing a decrease in photosystem II activity in the BSC chloroplasts, and in PEPCK subtypes the NADH that is released in the BSC mitochondria can be utilized to generate ATP. In some C4 plants, especially NADP-ME subtypes, another intercellular shuttle transfers NADPH and ATP from the MC to the BSC; 3PGA moves from the BSC to the MC and is reduced by phosphoglycerate kinase and NADP-glyceraldehyde-3-phosphate dehydrogenase to triose phosphates, which then move back to the BSC (shown in **A**).

Maier, A., Zell, M.B., and Maurino, V.G. (2011). Malate decarboxylases: evolution and roles of NAD(P)-ME isoforms in species performing C4 and C3 photosynthesis. Journal of Experimental Botany **62**, 3061-3069.

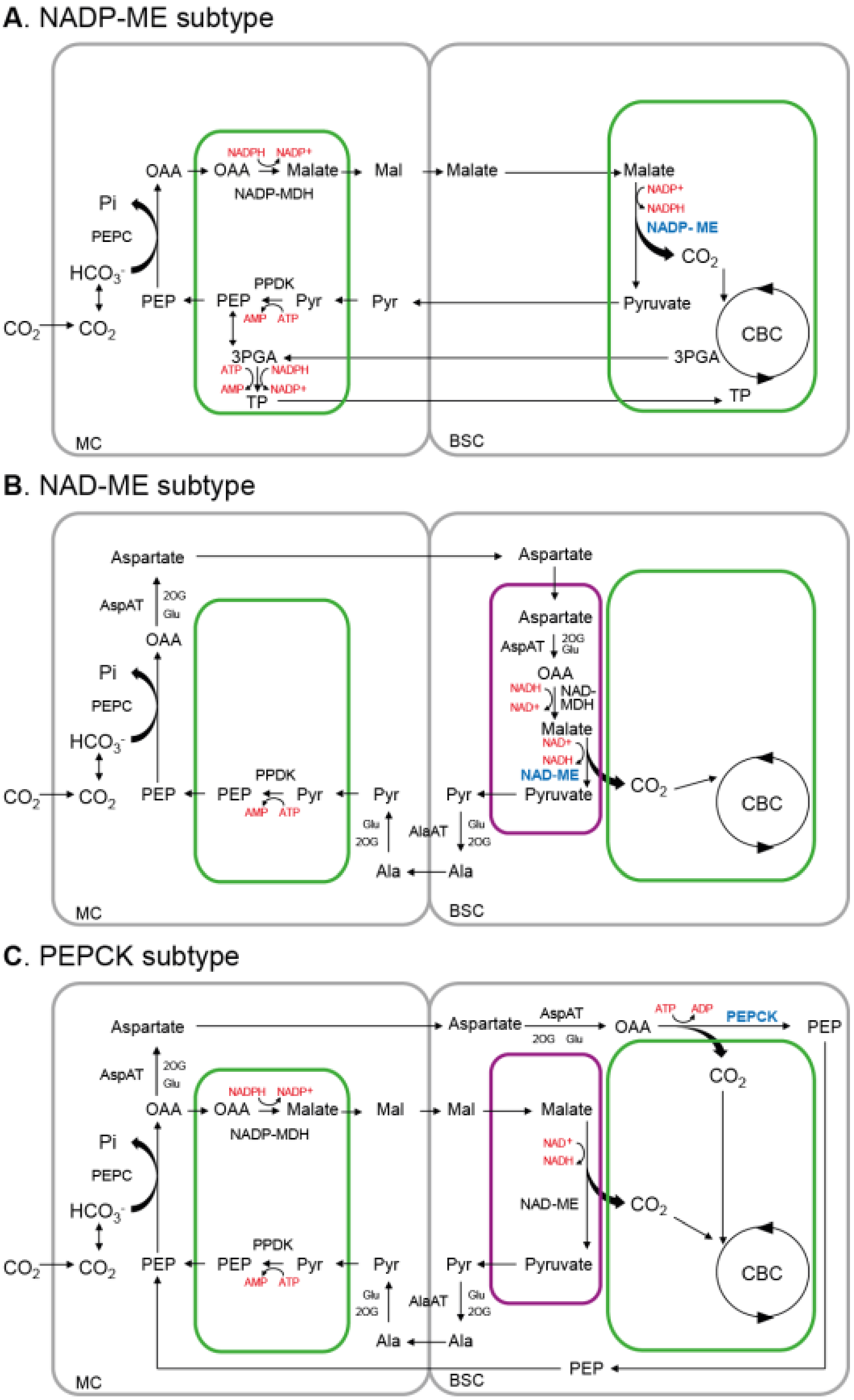

A. NADP-ME subtype
OAA
OAA
NADPH
NADP+
Malate
NADP-MDH
Mal
Malate
Malate
NADP+
NADPH
NADP- ME
CO2
Pi
PEPC
HCO3-
PEP
PEP
PPDK
Pyr
AMP
ATP
Pyr
Pyruvate
CBC
CO2
CO2
3PGA
3PGA
ATP
NADPH
AMP
NADP+
TP
TP
MC
BSC
B. NAD-ME subtype
Aspartate
Aspartate
AspAT
2OG
Glu
OAA
Pi
PEPC
HCO3-
CO2
CO2
PEP
PEP
PPDK
Pyr
AMP
ATP
Pyr
Aspartate
AspAT
2OG
Glu
OAA
NADH
NAD+
NAD-MDH
Malate
NAD+
NADH
NAD-ME
CO2
Pyr
Pyruvate
CBC
Glu
2OG
AlaAT
Glu
2OG
Ala
Ala
MC
BSC
C. PEPCK subtype
Aspartate
Aspartate
AspAT
2OG
Glu
OAA
ATP
ADP
PEPCK
PEP
AspAT
2OG
Glu
OAA
OAA
NADPH
NADP+
Malate
NADP-MDH
Mal
Mal
Malate
NAD+
NADH
NAD-ME
CO2
CO2
Pi
PEPC
HCO3-
CO2
CO2
PEP
PEP
PPDK
Pyr
AMP
ATP
Pyr
Pyr
Pyruvate
CBC
Glu
2OG
AlaAT
Glu
2OG
Ala
Ala
PEP
MC
BSC

Supplementary Figure S2. ^{13}C labeling systems and quenching procedures. **(A)**, **(B)** Setup for kinetic experiment. **(C)** Copper rod (precooled in liquid nitrogen) dropped in the custom-made labeling chamber containing a maize leaf. **(D)** Leaf disk obtained. **(E)** Setup for cell separation experiment. **(F)** Gas mixture containing $^{12}CO_2$ was removed from the labeling bag with a vacuum pump prior supplying a gas mixture containing $^{13}CO_2$. **(G)** Middle part of the leaf quenched between two metal blocks precooled by liquid nitrogen. **(H)** Leaf section obtained.

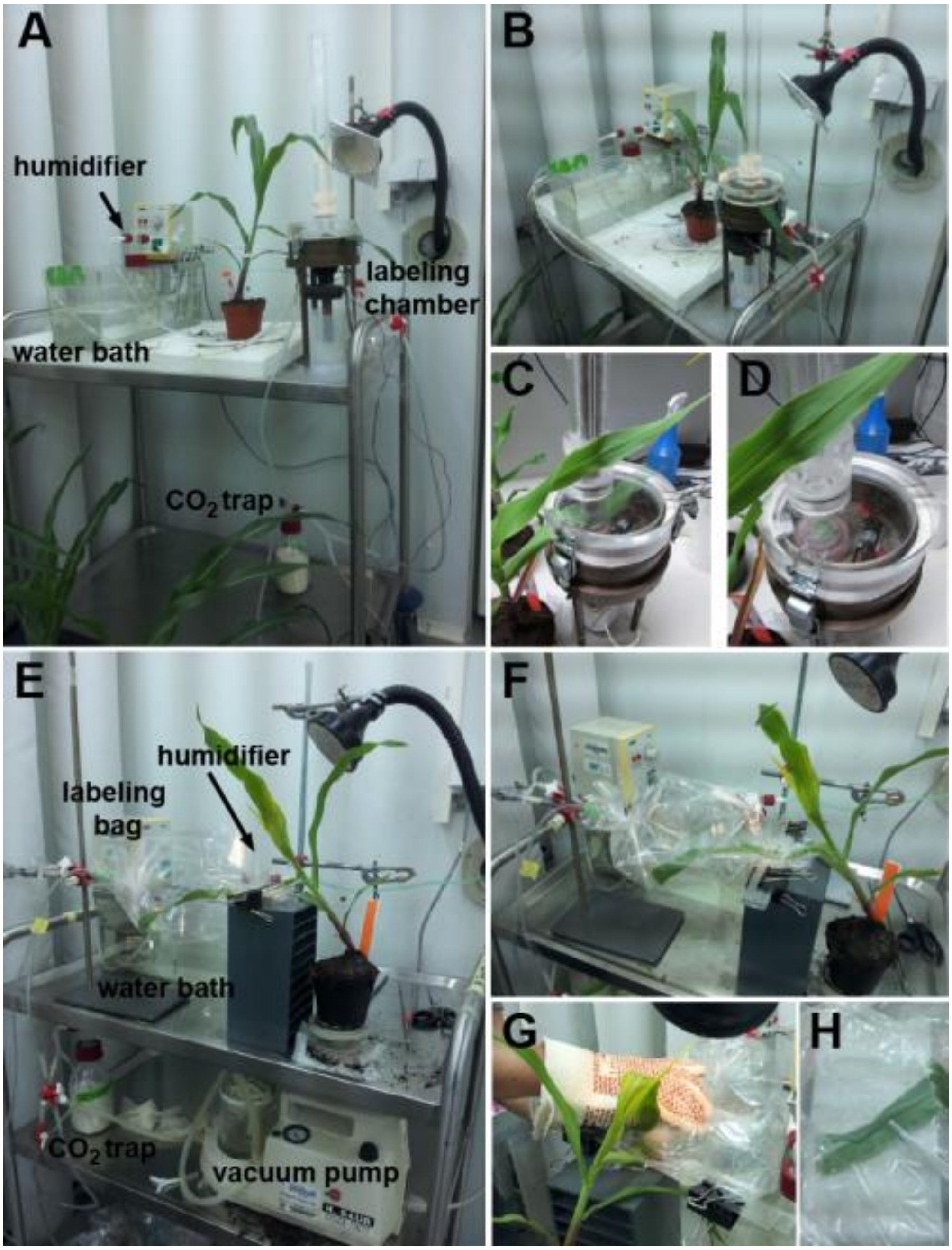

Supplementary Figure S3. Metabolic content of $^{13}CO_2$ labeled maize leaf during the day. For a given compound, amounts of all isotopomers were summed. Amounts are expressed as nmol g^{-1} FW except if an asterisk is present (µmol g^{-1} FW). The x-axis corresponds to the order in which samples were harvested during the day, spanning over a time period of 9 hour and half. For a better comprehension the times of sampling have been numbered and the corresponding average time of sampling (h:min after beginning of the light period) and the ^{13}C labeling duration are indicated. n.d. stands for not determined. PEP, phosphoenolpyruvate; 3PGA, 3-phosphoglyceric acid; DHAP, dihydroxyacetone phosphate; FBP, fructose-1,6-bisphosphate; F6P, fructose-6-phosphate; SBP, sedoheptulose-1,7-bisphosphate; S7P, sedoheptulose-7-phosphate; R5P, ribose-5-phosphate; Ru5P+Xu5P, ribulose-5-phosphate + xylulose-5-phosphate; RuBP, ribulose-1,5-bisphosphate; G6P, glucose-6-phosphate; G1P, glucose-1-phosphate; ADPG, ADP-glucose; UDPG, UDP-glucose; 2PG, 2-phosphoglycolate; 2OG, 2-oxoglutarate. The amounts of the unlabeled form and each ^{13}C-isotopomer are provided in Supplementary Table S3, and the summed amounts in Supplementary Table S4.

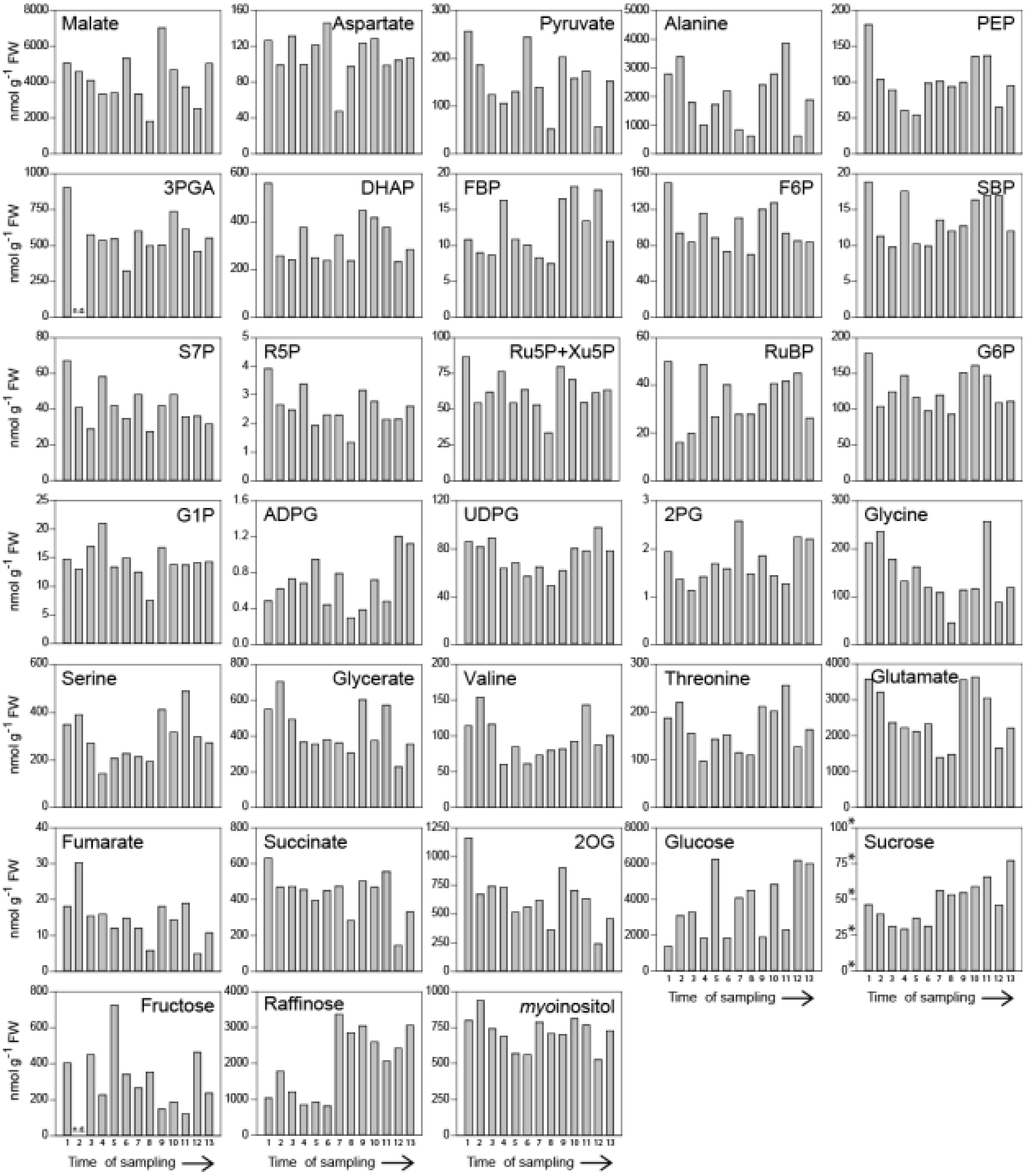

	1	2	3	4	5	6	7	8	9	10	11	12	13
Average sampling time	2:35	3:12	3:48	4:02	4:16	4:31	6:05	7:57	8:54	10:26	10:28	11:13	11:29
^{13}C labeling duration (s)	600	0	10	15	20	30	3600	2400	180	300	1200	50	60

Supplementary Figure S4. Overview of ^{13}C labeling kinetics from primary carbon metabolism by *k*-means clustering. Grey lines show the ^{13}C enrichment of individual metabolites and black lines show average ^{13}C enrichment of all metabolites in the cluster. The x-axis corresponds to the labeling time on a log_{10} scale. Data are provided in Supplementary Table S5.

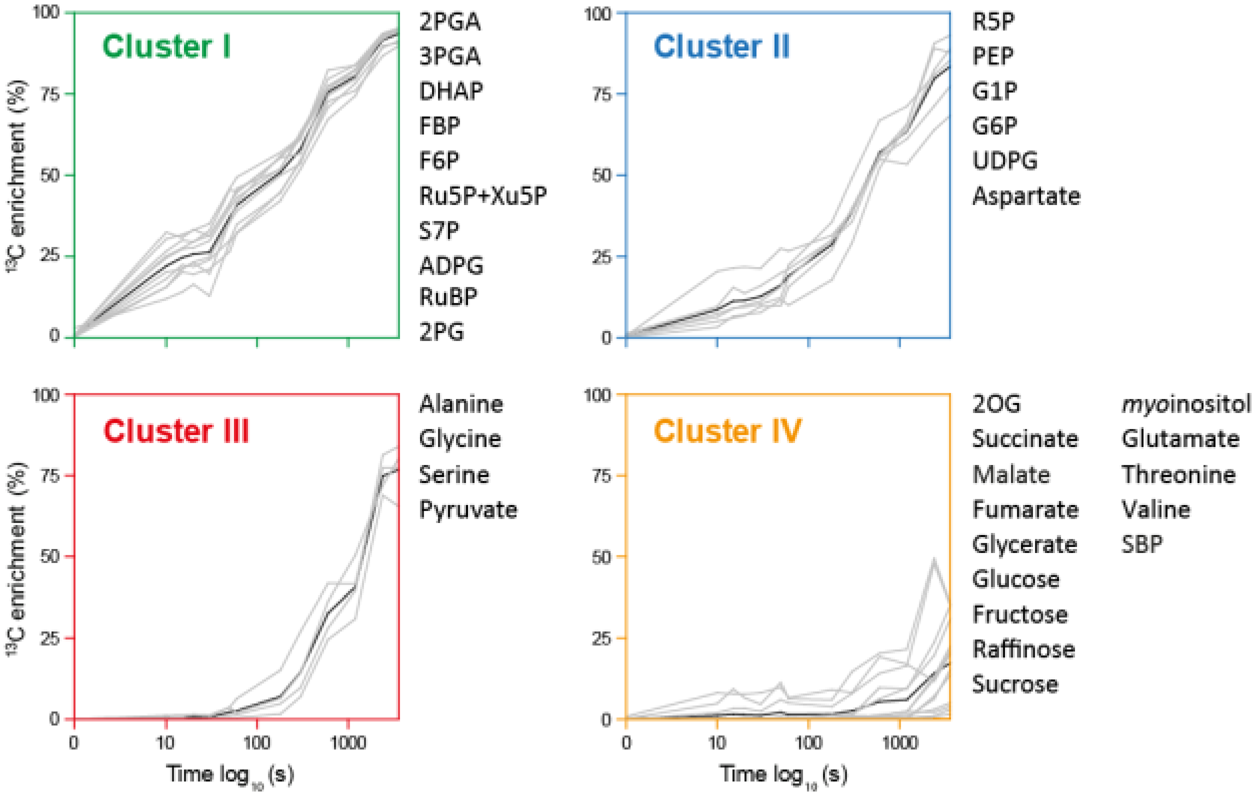

Supplementary Figure S5. Time-course of mass distribution of metabolites from CBC, starch and sucrose pathways. The relative abundance of each isotopomer (m_n) for a given metabolite is represented; *n* is the number of ^{13}C atoms incorporated. The graph for SBP[a] corresponds to the isotopomer distribution after correction for the inactive pool. The x-axis corresponds to the labeling time on a log_{10} scale. Data are presented in Supplementary Table S6.

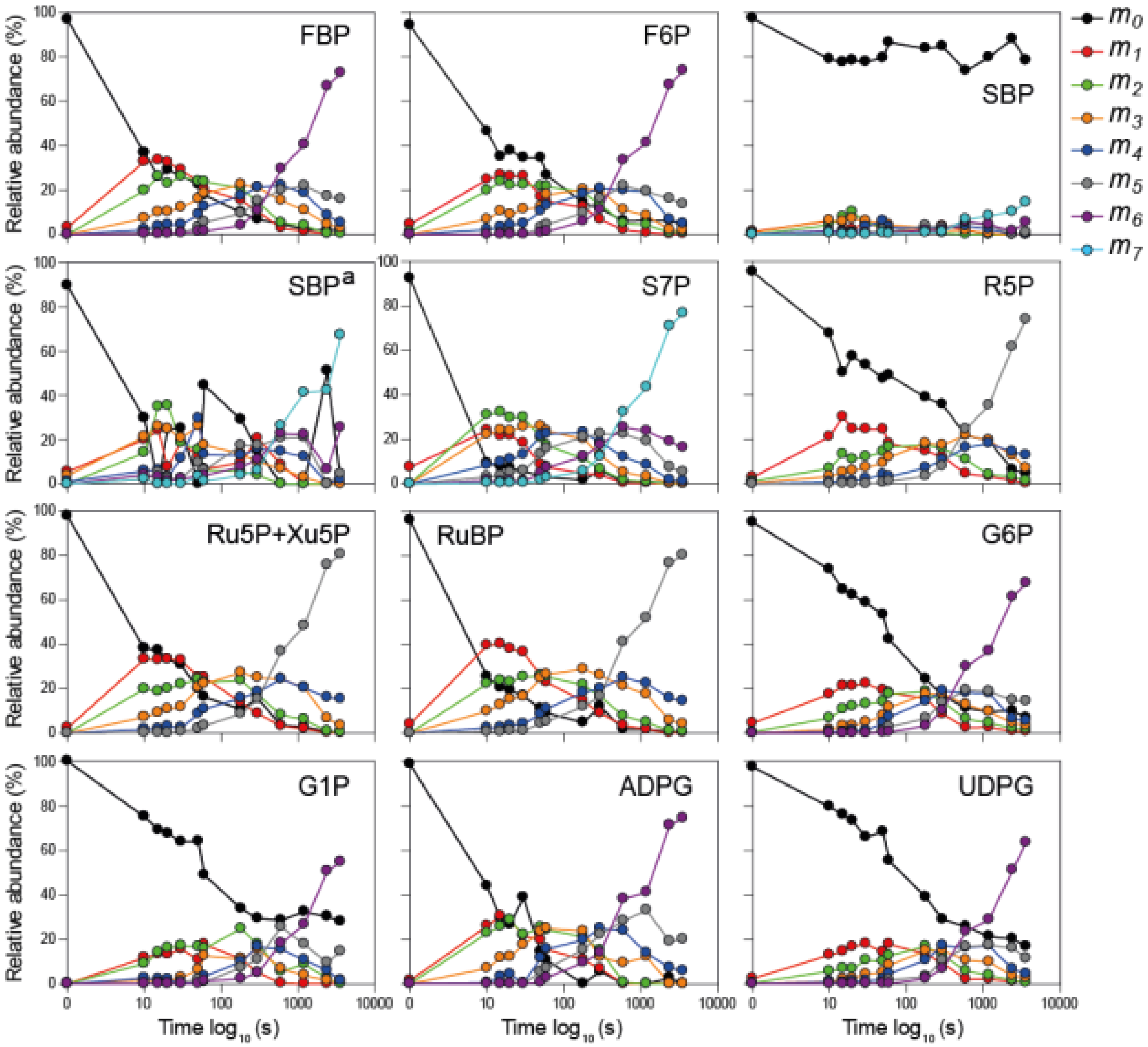

Supplementary Figure S6. Scheme of positional carbon incorporation in compounds from the CO_2 shuttle. (A) Carbon fixation in the CCS. $^{13}CO_2$ is incorporated into the C4 positon of OAA and passed on to the C4 positons of malate and aspartate, and ^{13}C is released from the C4 position by decarboxylation reactions in the BSC. Consequently no label is introduced into the 3-carbon products of the decarboxylation reaction like pyruvate and alanine **(B)** Carbon transfer between 3PGA and the CCS. Once ^{13}C is incorporated in the CBC label is randomized due to rapid turnover of the CBC. 3PGA moves to the MC and exchanges via 2PGA with the PEP formed by PPDK, leading to ^{13}C being incorporated into positions C1-3 of OAA, malate and aspartate, and thence into pyruvate and alanine.

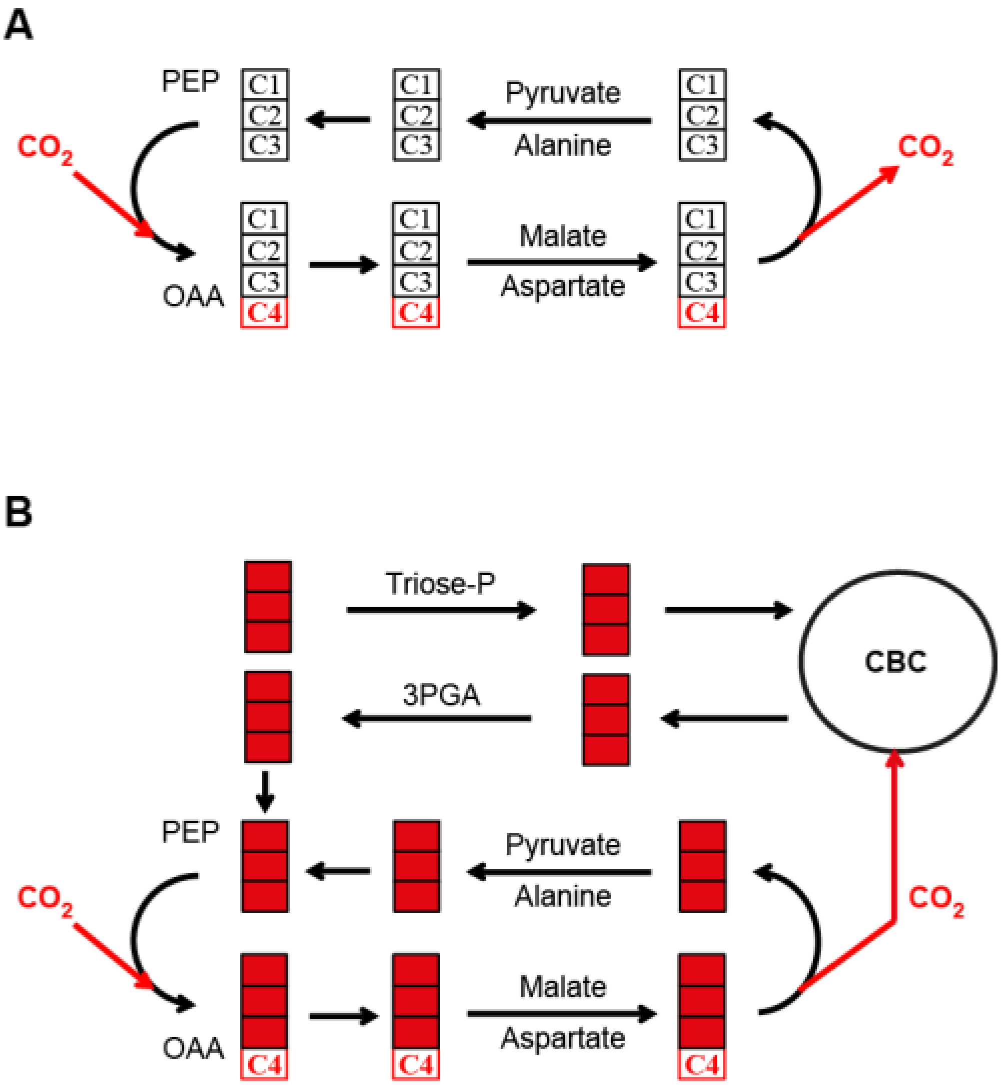

Supplementary Figure S7. Time-course of mass distribution of metabolites in malate and aspartate during a chase. After 60 min labeling (time 0), $^{13}CO_2$ was replaced by $^{12}CO_2$ and chase performed for 5 and 20 min. The relative abundance of each isotopomer (m_n) for a given metabolite is represented; n is the number of ^{13}C atoms incorporated. The graph for malate[a] corresponds to the isotopomer distribution after correction for the inactive pool. The x-axis corresponds to the labeling time on a log_{10} scale. Data are presented in Supplementary Table S6.

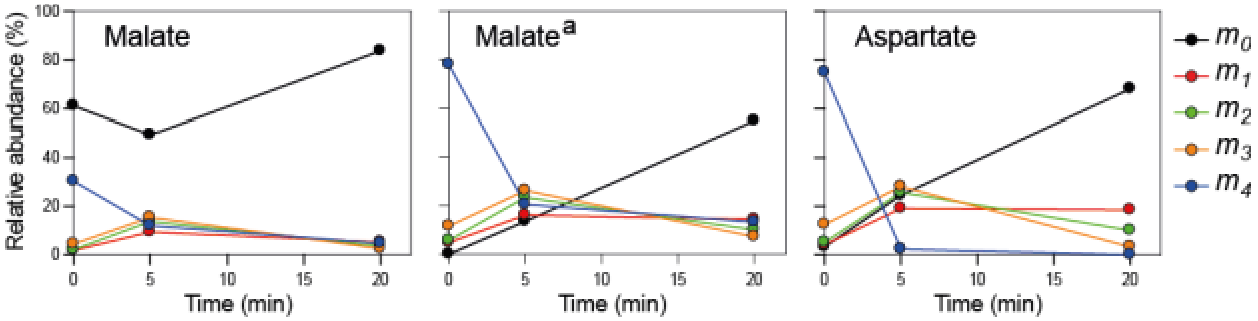

Supplementary Figure S8. Regression plots of ^{13}C amounts calculated with two approaches. ^{13}C amounts (natom ^{13}C equivalents g^{-1} FW) in metabolites were estimated by multiplying the number of ^{13}C atoms per isotopomer by the corresponding isotopomer amount, and then summing the results. In approach A, isotopomer amounts were calculated by multiplying isotopomer abundance by the average pool size of the intermediate (or the active pool size as determined at 60 min for malate). Approach B used the isotopomer amounts determined in each sample. Amounts for malate, aspartate, malate minus C4 position and aspartate minus C4 position were calculated with both approaches until time point 180 sec (Supplementary Table S8) and results obtained with approach A (y-axis) were plotted against results obtained with approach B (x-axis). Slope and R^2 for each regression line are indicated.

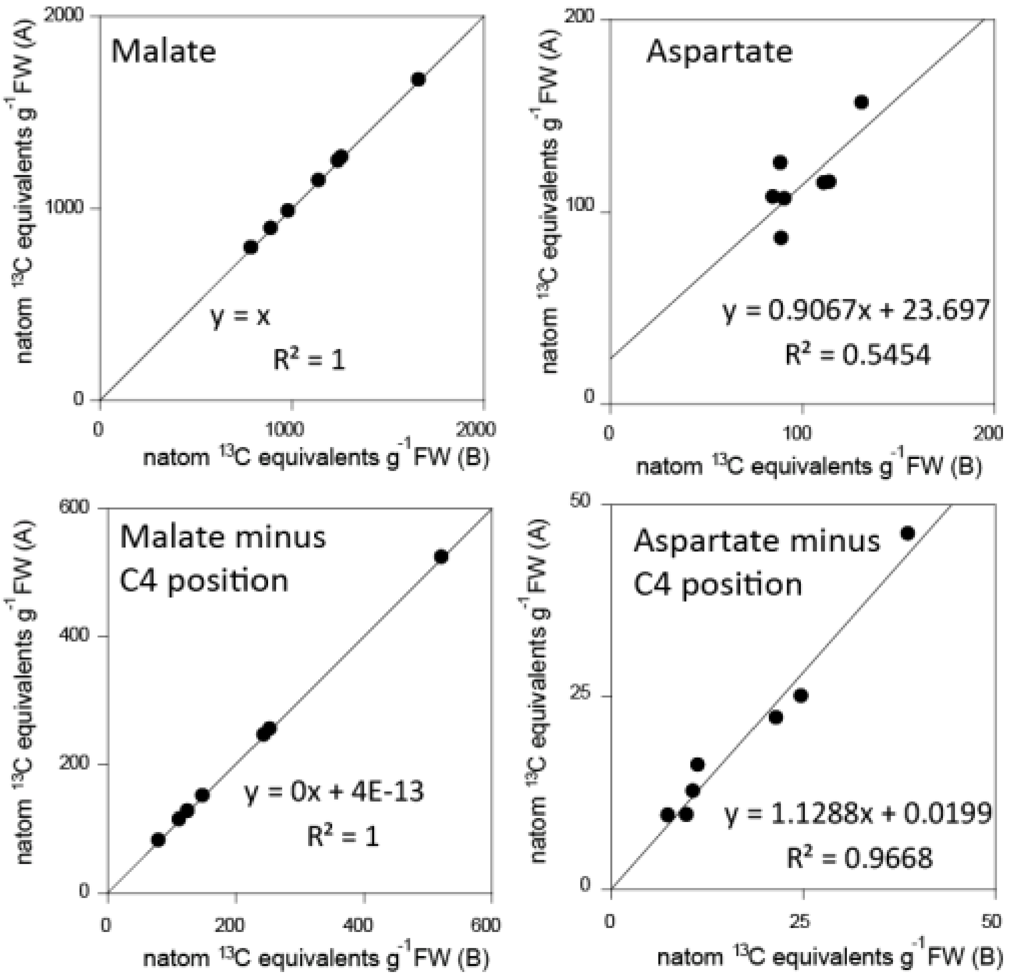

Supplementary Figure S9. Time-course of mass distribution of photorespiration cycle intermediates, amino acids, organic acids and sugars. The relative abundance of each isotopomer (m_n) for a given metabolite is represented; *n* is the number of ^{13}C atoms incorporated. The x-axis corresponds to the labeling time on a log_{10} scale. Data are provided in Supplementary Table S6.

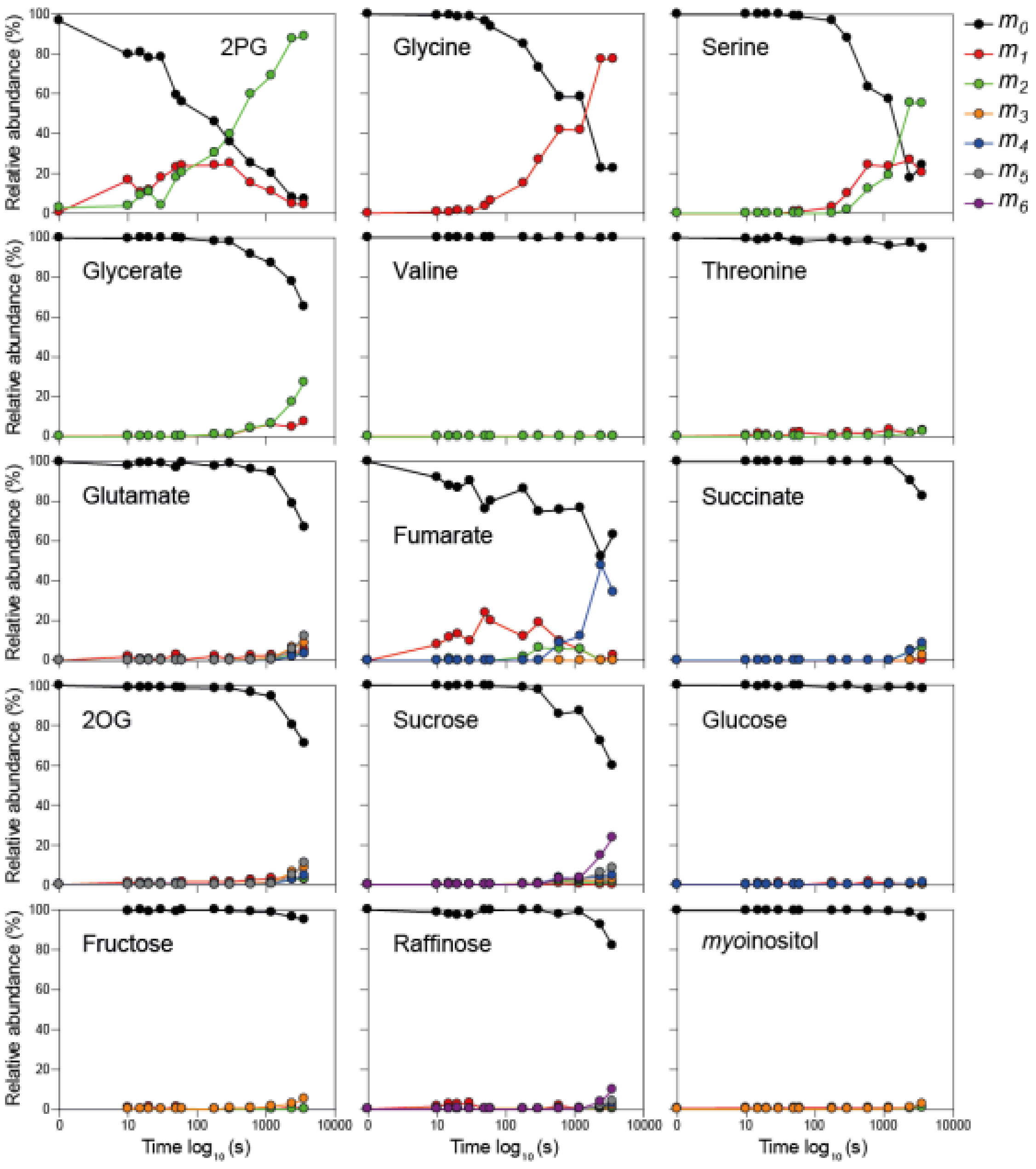

Supplementary Figure S10. Amounts of photorespiratory intermediates in *Arabidopsis thaliana* labeled with $^{13}CO_2$ under 21 and 2% O_2 . Values are means ± SD (*n* = between 3 and 13). Data are provided in Supplementary Table S10. Significant differences (according to Student's t-test) of values at each time point obtained at 2% O_2 from all values obtained in unlabeled Arabidopsis under 21% O_2 (indicated as "all", dark grey) are indicated by asterisks (*P < 0.05, **P < 0.01 and ***P < 0.001).

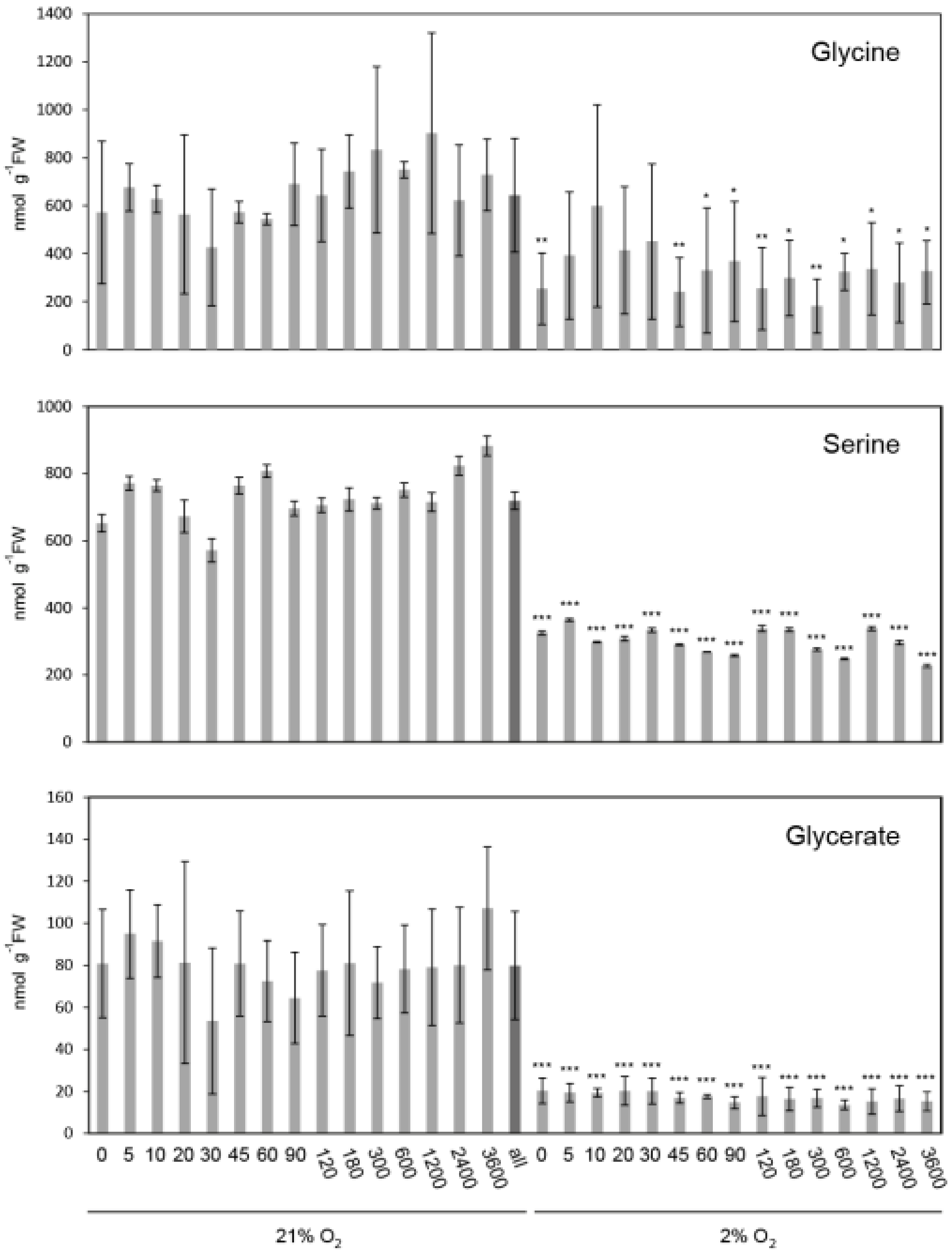

Supplementary Figure S11. Time-course of mass distribution of glycine in *Arabidopsis thaliana* labeled with $^{13}CO_2$ under 21 and 2% O_2. The relative abundance of each isotopomer (m_n) for a given metabolite is represented; n is the number of ^{13}C atoms incorporated. Values are means ± SD (n = between 3 and 13). Data are provided in Supplementary Table S10.

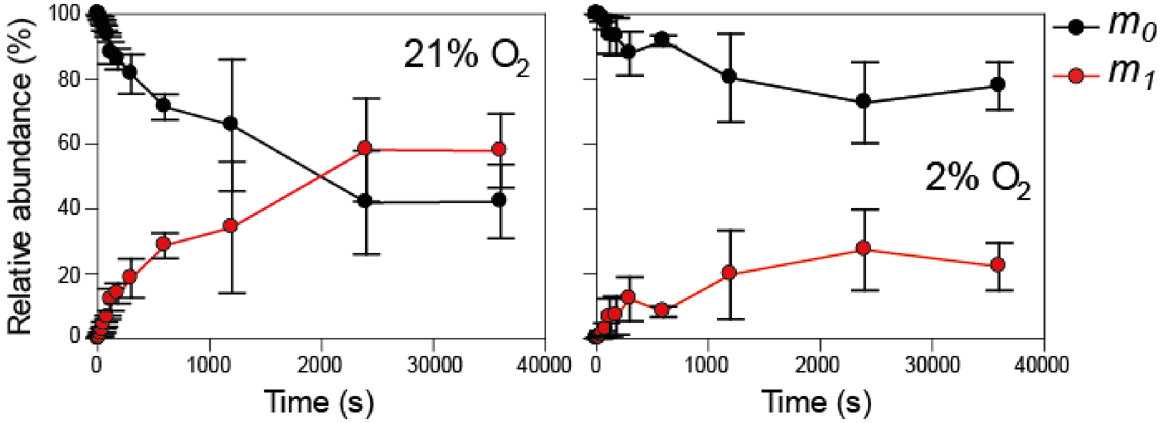

Supplementary Figure S12 Regression plots of ^{13}C enrichments from the kinetic experiment and material for cell separation. The ^{13}C enrichments of 33 shared metabolites between both experiments were plotted against each other, using average values (n = 3) from the material for cell separation as x-axis and enrichments at the different kinetic time points for the y-axis. Slope and R^2 for each regression line are indicated. Data used are presented in Supplementary Table S5. Corrected ^{13}C enrichments were used for malate, alanine, SBP, G1P, glycine, serine and fumarate (as indicated with [a] in Supplementary Table S5).

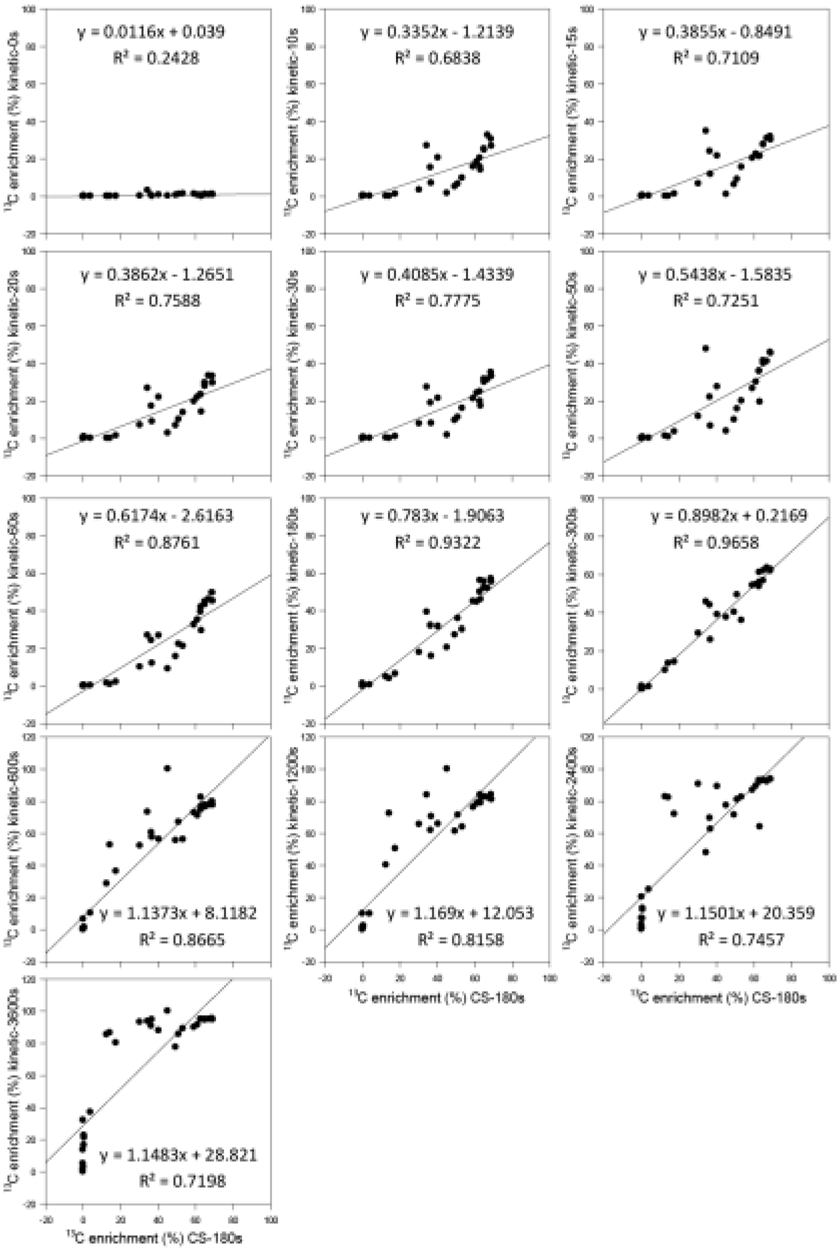

Supplement: Supplementary_Figures_S1_S12 [file erw414_suppl_supplementary_figures_s1_s12.pdf]
